# Lessons for Oral Bioavailability: How Conformationally Flexible Cyclic Peptides Enter and Cross Lipid Membranes

**DOI:** 10.1021/acs.jmedchem.2c01837

**Published:** 2023-02-10

**Authors:** Stephanie M. Linker, Christian Schellhaas, Anna S. Kamenik, Mac M. Veldhuizen, Franz Waibl, Hans-Jörg Roth, Marianne Fouché, Stephane Rodde, Sereina Riniker

**Affiliations:** † Department of Chemistry and Applied Biosciences, ETH Zürich, Vladimir-Prelog-Weg 2, 8093 Zürich, Switzerland; ‡ Novartis Institutes for BioMedical Research, Novartis Pharma AG, Novartis Campus, 4056 Basel, Switzerland

## Abstract

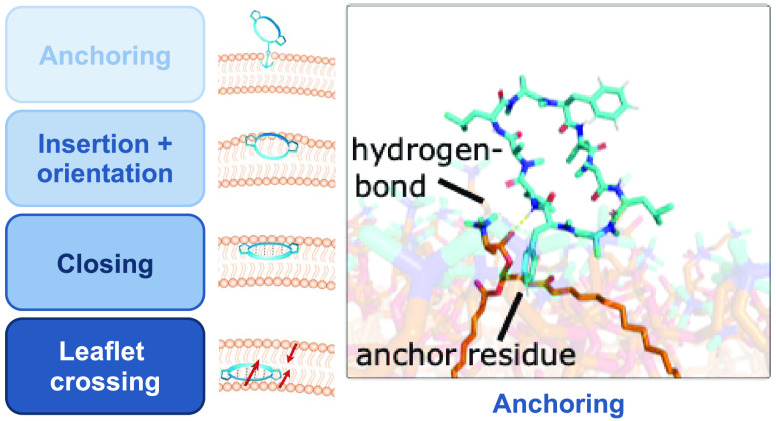

Cyclic peptides extend the druggable target space due to their size, flexibility, and hydrogen-bonding capacity. However, these properties impact also their passive membrane permeability. As the “journey” through membranes cannot be monitored experimentally, little is known about the underlying process, which hinders rational design. Here, we use molecular simulations to uncover how cyclic peptides permeate a membrane. We show that side chains can act as “molecular anchors”, establishing the first contact with the membrane and enabling insertion. Once inside, the peptides are positioned between headgroups and lipid tails—a unique polar/apolar interface. Only one of two distinct orientations at this interface allows for the formation of the permeable “closed” conformation. In the closed conformation, the peptide crosses to the lower leaflet via another “anchoring” and flipping mechanism. Our findings provide atomistic insights into the permeation process of flexible cyclic peptides and reveal design considerations for each step of the process.

## Introduction

Macrocyclic compounds like cyclic peptides represent promising candidates to address difficult drug targets.^1−7^ Traditional small-molecule drugs typically show sufficient binding affinity only for relatively deep and narrow protein binding sites.^8,9^ Consequently, this limits their target space to an estimated 10–15% of the human proteome.^10−12^ In comparison to small molecules, macrocyclic compounds hold the potential to vastly extend the scope of druggable proteins. Due to their ability to target larger binding sites with flat profiles,^4,13−17^ like protein–protein interaction (PPI) interfaces,^18−20^ they can lead to new therapeutics for currently untreatable diseases. However, high binding affinity to a target is not enough for a molecule to be pharmaceutically relevant. One crucial aspect in drug design is drug delivery, where passive membrane permeability is important for oral bioavailability of drugs. Key steps of drug delivery like gastrointestinal absorption and passing the portal venous system^21,22^ predominantly rely on trans-cellular diffusion.^23^ Moreover, a majority of drugs with intracellular targets also pass the final cell-membrane barrier via passive diffusion.^24^ Macrocycles often possess high molecular weights and low lipophilicity that are associated with low passive permeability,^25^ hampering the therapeutic applications of macrocyclic compounds.^25−28^

Cyclic peptides are macrocycles that are composed of amino acids.^29^ In comparison to their linear counterparts, they are associated with better passive cell membrane permeability and metabolic stability.^26,29,30^ Interestingly, there are examples of cyclic peptides that can be administered orally,^31,32^ despite violating conventional drug-likeliness rules based on their increased molecular weight and high number of hydrogen-bonding atoms.^24,25,28,33,34^ Nonetheless, designing orally bioavailable cyclic peptides with simultaneous high binding affinity to the target has been difficult so far, and is often only achieved via a tedious trial-and-error process.^35−38^ Addressing this issue, previous studies have substantially advanced our understanding of the structure–permeability relationship of cyclic peptides, and thus provide guidance for their design as therapeutics with oral bioavailability.^25,30,39−46^*N*-Methylation of the peptide backbone,^47−49^ changes of stereocenters,^50,51^ tuning the amphiphilicity,^52^ and side-chain modifications^53,54^ were identified as membrane permeability factors. The effects of these modifications are unfortunately nonlinear and highly site-dependent.^53,55−59^ Even small structural modifications can lead to global conformational rearrangements and thus change the physicochemical properties and membrane permeability of a compound.^6,60,61^ Coherently, it has been shown that the conformational behavior of a cyclic peptide in different environments is particularly impactful for passive permeability.^62−70^

Cyclic peptides with so-called “chameleonic” behavior are able to adapt to their environment and adopt different conformational states that exhibit varying lipophilic properties.^62,63,65,68,71−74^ These conformational states can often be classified by their number of intramolecular hydrogen bonds: In so-called “open” conformations, hydrogen-bonding atoms are exposed, allowing for formation of favorable contacts with polar solvent molecules (e.g., in the bloodstream or the cytosol). In the so-called “closed” conformation, intramolecular hydrogen bonds are formed, which leads to a less polar surface area and a lower desolvation energy when entering apolar environments like the cell membrane interior. Intuitively, this behavior of chameleonic cyclic peptides is beneficial for oral bioavailability, as good permeability is combined with good solubility.^75^

It is generally assumed that the closed conformation is the main permeable species.^53,63−65,67,76^ However, little is known about the structural origin of the chameleonic properties, let alone the mechanistic details of the path of a chameleonic cyclic peptide through the cell membrane with respect to its conformational behavior. While the composition (amino acid sequence), size, and hydrophobic surface have been identified as important determinants of chameleonic conformational behavior,^25,55,56,67,77^ their mechanistic interplay is complex and not yet understood well enough to elucidate structure–permeability relationships. Addressing this challenge, molecular dynamics (MD) simulations can serve as a computational microscope to track the pathway and conformational dynamics of cyclic peptides in a time and spatial resolution that is not (yet) feasible with current experimental techniques. In combination with experimental data (e.g., PAMPA membrane permeability coefficients^78^), this enables the development of a holistic view of the passive permeability of cyclic peptides.^70^

In this study, we perform extensive atomistic MD simulations of a series of eight cyclic decapeptides that show complex internal conformational dynamics in a 1-palmitoyl-2-oleoyl-phosphatidylcholine (POPC) bilayer system to decipher their pathway through the membrane and rationalize the relationship between structure and passive membrane permeability. Previous approaches have focused on simulations either in homogeneous solvents,^53,67^ at very high temperatures,^79^ or under application of steered pulling forces.^46,80^ We report an alternative sampling strategy where insights from unbiased MD simulations are used to seed biased simulations that allow for a stepwise enrichment of key events across the permeation pathway. Note that this strategy enabled us to describe the membrane crossing pathway in an unbiased manner at room temperature, avoiding artifacts like pore formation or other distortions of the POPC bilayer.

**Figure 1:**
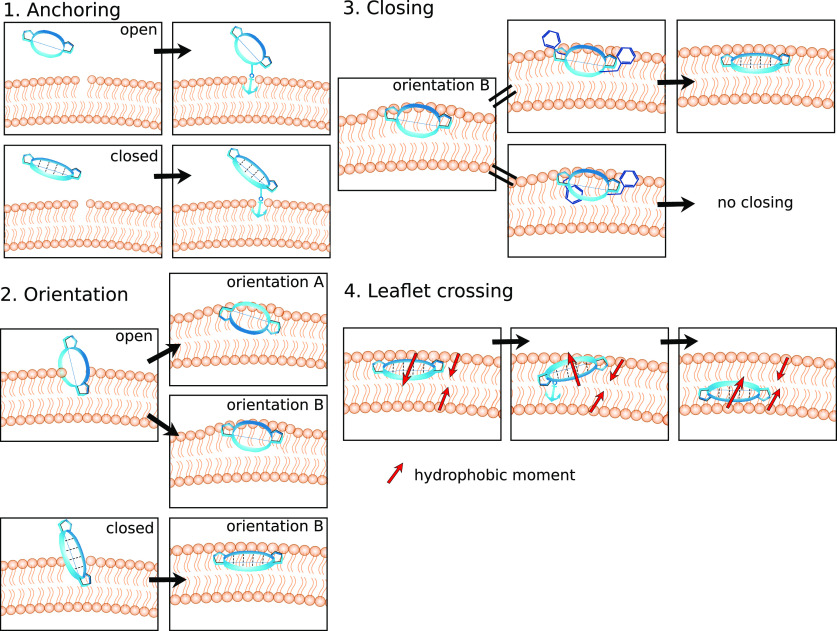
Summary of the four steps for the passive membrane permeation of conformationally flexible cyclic peptides. The peptide is shown in blue and the membrane in orange. Peptides in the closed conformation are indicated by their intramolecular hydrogen bonds (dashed lines). Side-chain residues that anchor the peptide in the membrane are depicted with an anchor symbol. The hydrophobic moment is pointing from the polar to the apolar part of a molecule.

Our key findings are summarized in Figure 1, highlighting the four steps, which we identified, for the passive membrane permeation of conformationally flexible cyclic peptides. First, specific side-chain residues can act as “molecular anchors”, which establish the contact between a cyclic peptide and a membrane before insertion. Second, the peptide positions itself directly at the interface between the polar headgroups and the apolar tail region. There, the cyclic peptides show a preference for one of two distinct orientations. Third, we observed conformational interconversion into the permeable closed state from only one of these orientations. Last, only this closed conformation can detach from the polar/apolar interface and diffuse across the lipid membrane leaflets, which again requires a unique anchoring and flipping mechanism. Based on these steps, we identified design considerations and opportunities that may improve the development of cyclic peptides with oral bioavailability.

## Results and Discussion

**Figure 2:**
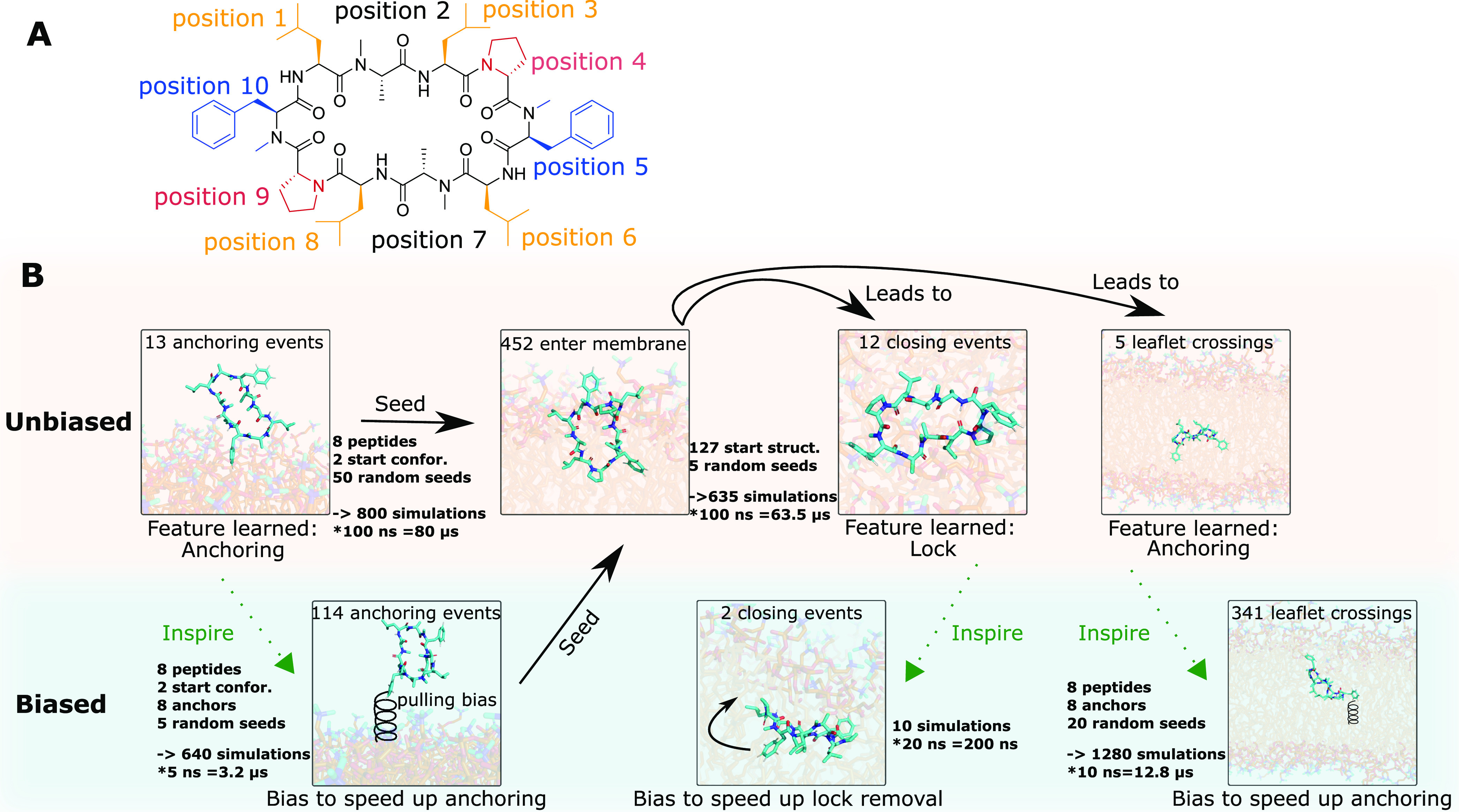
(A) Backbone scaffold and amino acid composition of the cyclic decapeptide (CDP) series used in this work. The colored residues were systematically replaced according to Table 1. The backbone scaffold was reported by Fouché et al.^81,82^ and is kept constant. (B) Schematic workflow showing the different conditions, total simulation time, and number of observed events. Unbiased MD simulations were used to elucidate the membrane permeation pathway of CDPs. Biasing along steps of this pathway was used to enrich sampling and to obtain starting structures for new unbiased simulations.

To investigate the permeability pathway of flexible cyclic peptides, we performed extensive atomistic MD simulations of a series of cyclic decapeptides (CDPs) at room temperature. The backbone scaffold and *N*-methylation pattern of the CDPs was introduced by Fouché et al.^81,82^ and kept constant. The variable amino acids in this series are highlighted in color in Figure 2A and listed in Table 1. The peptides are characterized by two β-strands (residues 1–3 and 6–8) and two β-turns (residues at positions 4, 5 and 9, 10). In the closed conformation, four intramolecular hydrogen bonds are formed that shield the polar groups of the CDP from the environment.^67^ The NMR solution structures and passive permeability data for the CDPs were taken from the literature.^53,67,81^

**Table 1:** Amino Acid Composition of the CDPs Used in This Study^a^

CDP	pos. 1	pos. 3	pos. 4	pos. 5	pos. 6	pos. 8	pos. 9	pos. 10	PAMPA log *P*_e_
**1**	Leu	Leu	d-Ala	M-Phe	Leu	Leu	d-Ala	M-Phe	–5.9
**2**	Leu	Leu	d-Ala	M-Ala	Leu	Leu	d-Ala	M-Ala	–4.0
**3**	Leu	Leu	d-Pro	M-Phe	Leu	Leu	d-Pro	M-Phe	–5.3
**4**	Leu	Leu	d-Pro	M-Phe	Leu	Leu	d-Pro	M-Ala	–4.2
**5**	Leu	Leu	d-Pro	M-Ala	Leu	Leu	d-Pro	M-Ala	–4.6
**6**	Ala	Leu	d-Pro	M-Phe	Ala	Leu	d-Pro	M-Phe	–4.1
**7**	Ala	Ala	d-Pro	M-Phe	Leu	Leu	d-Pro	M-Phe	–4.4
**8**	Ala	Ala	d-Pro	M-Phe	Ala	Ala	d-Pro	M-Phe	–6.4

a d-Amino acids are marked with the letter d, methylated amino acids are marked with the letter M. The amino acids at position 2 and 7 (M-Ala) were kept constant. The parallel artificial membrane permeation assay (PAMPA) coefficients were taken from ref (69). CDP **1** and **8** with log *P*_e_ < −5.5 can be considered non-permeable.

The permeation process of the CDPs was tracked through cycles of unbiased and biased simulations. Unbiased simulations were used to learn the features important for permeability. This knowledge was utilized to define the collective variable for biasing. From the end states of the biased simulations, new unbiased simulations were started (see Figure 2B). Unless clearly marked otherwise, all results and conclusions in this paper were drawn from the analysis of the unbiased simulations.

### Cyclic Peptides Enter Lipid Membranes Using Anchor Residues

**Figure 3:**
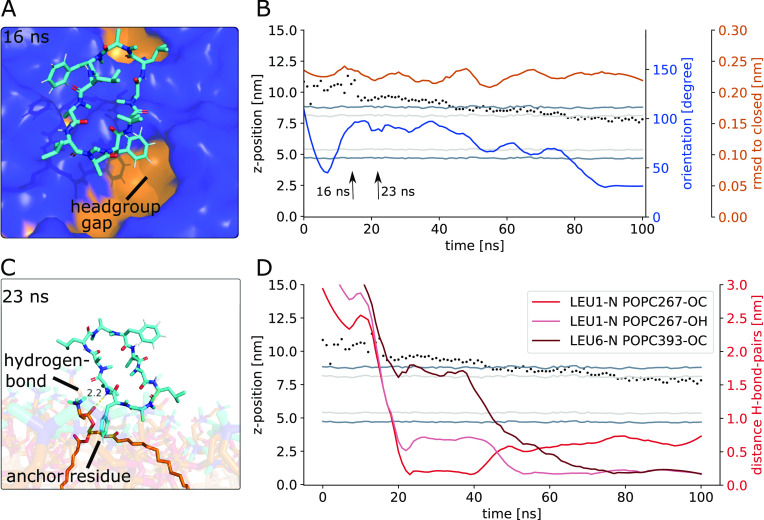
(A) Snapshot of CDP **1** directly before anchoring to the membrane. The atoms of the POPC headgroups are colored in blue; the atoms of the tails are colored in orange. Thermal fluctuations of the lipids can lead to temporary headgroup gaps and the apolar tails underneath become exposed (in this snapshot the gaps manifest as orange patches). If the CDP is close to such a transient gap, its apolar side chains can “anchor” to the lipid membrane. (B) Trajectory of CDP **1** entering a membrane. The *z*-position of the CDP is indicated with black dots. The position of the headgroup and tail region are indicated with dark gray and light gray lines, respectively. The angle between the normal vectors of the peptide and the membrane is shown in blue. The RMSD with respect to the closed conformation of the CDP is shown in orange. (C) Snapshot of the CDP while anchoring to the membrane. A hydrogen bond between the CDP backbone and the polar headgroup atoms can stabilize the anchoring. (D) During the anchoring process, three consecutive hydrogen bonds are formed. The distance between the hydrogen-bond pairs are shown in red. The *z*-positions of the CDP and the membrane are shown as in panel B.

The simulations of the CDPs started in the aqueous phase at a distance of around 3 nm from the membrane and were allowed to freely diffuse through the aqueous phase. Within the sampled 100 ns per simulation, only a small fraction of the simulations resulted in peptide–membrane contacts (i.e., 13 out of 800). Interestingly, all these interactions followed the same mechanism: First, transient and small gaps between the headgroups of the membrane formed due to thermal fluctuations. During this short time span, the apolar lipid tails are exposed to the aqueous phase. Figure 3A visualizes the opening of these gaps, where the solvent-accessible surface of the membrane is color coded as headgroups (blue) or tail region (orange). If, by chance, the CDP is in close proximity to such a gap, its apolar residues can interact with the exposed lipid tails and the peptide is stabilized at the water–membrane interface. Hence, we find that an apolar side chain acting as a molecular anchor is a key feature for this contact initiating step (see Figure 3C). However, as the headgroup gaps are short-lived (<20 ps)^83^ and small (for a size distribution see SI Figure S1), contact formation happens on average only once per 6 μs simulation time. The area per lipid is not significantly perturbed upon entry of the peptide (see SI Figure S2).

Figure 3B depicts a simulation of a CDP anchoring and entering the lipid bilayer in more detail. To better track the position of the peptide, the dark gray points indicate the position of the upper headgroup region and the light gray points indicate the beginning of the apolar tails as defined in the Data Analysis section. A movie of an anchoring event is also available in the SI. For the first 16 ns, the peptide diffuses freely in the aqueous phase. Then, the first membrane contact is established. The peptide stays anchored to the membrane for 26 ns. In this anchored position, the angle between the normal vectors of the peptide and the membrane is around 90° (see blue line). Thus, the peptide is oriented perpendicular to the membrane. At around 42 ns, the peptide slowly penetrates deeper into the membrane. This causes no significant perturbation in the area per lipid (see SI Figure S2). The peptide first moves through the headgroup region in its initial nearly perpendicular orientation. Then, the peptide gradually rotates to be parallel with the membrane plane as it continues the permeation process. Toward the end of the simulation (100 ns), the peptide is located directly at the interface between the polar headgroups and apolar tails (see also SI Figure S3). As indicated by the RMSD plotted in orange in Figure 3B, in this particular simulation CDP **1** inserts into the membrane in an open conformation. While we also observed anchoring events in the closed conformation for other CDPs (see SI Figure S3), there was a strong imbalance toward anchoring in an open conformation (11 out of 13 unbiased anchoring events, see SI Table S1). This was surprising because the closed and open conformations were equally represented in our starting structures and most of the CDPs have a significant equilibrium population of the closed conformation in water.^53,69^ More so, in four simulations we observed CDPs that started in the closed conformation and opened prior to entering the membrane.

Figure 3C shows a snapshot of the peptide (here CDP **1**) anchored at the membrane. In this particular simulation, a phenylalanine side chain acts as the “anchor”. In addition to phenylalanine, we observed that also the leucine and proline side chains are possible membrane anchors. Here, the phenylalanine “pulls” the peptide toward the membrane through favorable contact with the apolar tail region. In addition, this anchored structure is stabilized by a hydrogen bond between a POPC headgroup and the backbone of leucine at position 1. As indicated in Figure 3D, three hydrogen bonds are consecutively formed between the peptide and the membrane in the course of the anchoring and entering process. The first hydrogen bond, as described above, is formed between the phosphate group and leucine 1. As the peptide penetrates deeper into the membrane (at approximately 50 ns), the initial hydrogen bond is replaced by a new hydrogen bond between the same peptide residue and the ester oxygen of the lipid tail. Later (at approximately 75 ns), an additional hydrogen bond between the backbone nitrogen of leucine 6 and the phosphate group of another POPC molecule is formed. Stabilizing hydrogen bonds were observed for all anchoring events in open conformations. In contrast to the open conformations, the closed conformation is characterized by four intramolecular hydrogen bonds. Hence, such stabilizing interactions require a transitional breaking of an intramolecular hydrogen bond in favor of membrane–peptide interactions. We observed such transient hydrogen bond breaking and forming in both closed anchoring events. The associated energy barrier might be one reason that membrane contacts in the open conformations are more prevalent in our simulations compared to contacts in the closed conformation.

The unbiased simulations described so far are an adequate simulation approach to answer the question of how cyclic peptides enter lipid membranes with only limited bias through the simulation setup. However, the high computational cost associated with these simulations prevented us from obtaining sufficient statistics to derive hypotheses for the rational design of permeable cyclic peptides. Nevertheless, the information gathered in the unbiased simulation provided an ideal starting point for enhanced sampling, in our case pulling simulations. The basic idea in pulling simulations is to introduce a biasing potential along a chosen “reaction coordinate”. With this technique, we could significantly enhance the occurrence of anchoring events within feasible computational time. Based on the mechanism observed in the unbiased simulations, we applied a weak pulling force on each of the potential anchor residues and pulled toward the membrane center (for details see the Biased simulations section and Figure 2B). Additionally, we performed simulations where we applied the pulling force on the center-of-mass (COM) of the peptide (see SI Figure S4). However, only when the bias is applied to the potential anchor residues we observed that the peptides do anchor and subsequently enter the membrane. This emphasizes the importance of selecting an appropriate reaction coordinate for biased simulations. Hence, in our case insights on the permeation mechanism from the unbiased simulations were vital to be able to bias effectively but cautiously, with minimal distortion of the system. By applying the bias, anchoring events occurred on average every 30 ns, which corresponds to a 200-fold speed-up.

**Figure 4:**
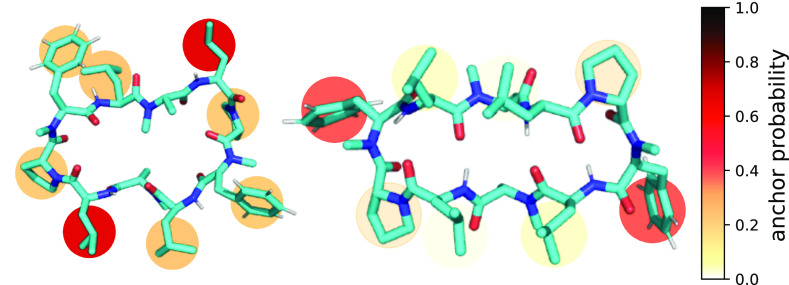
“Anchor quality” of the different side chains for the CDPs in open (left) and closed (right) conformations. The anchor probability was determined as the fraction of successful anchoring events after pulling the CDPs on that respective side chain toward the membrane. The probabilities are averaged over the eight CDPs.

**Figure 5:**
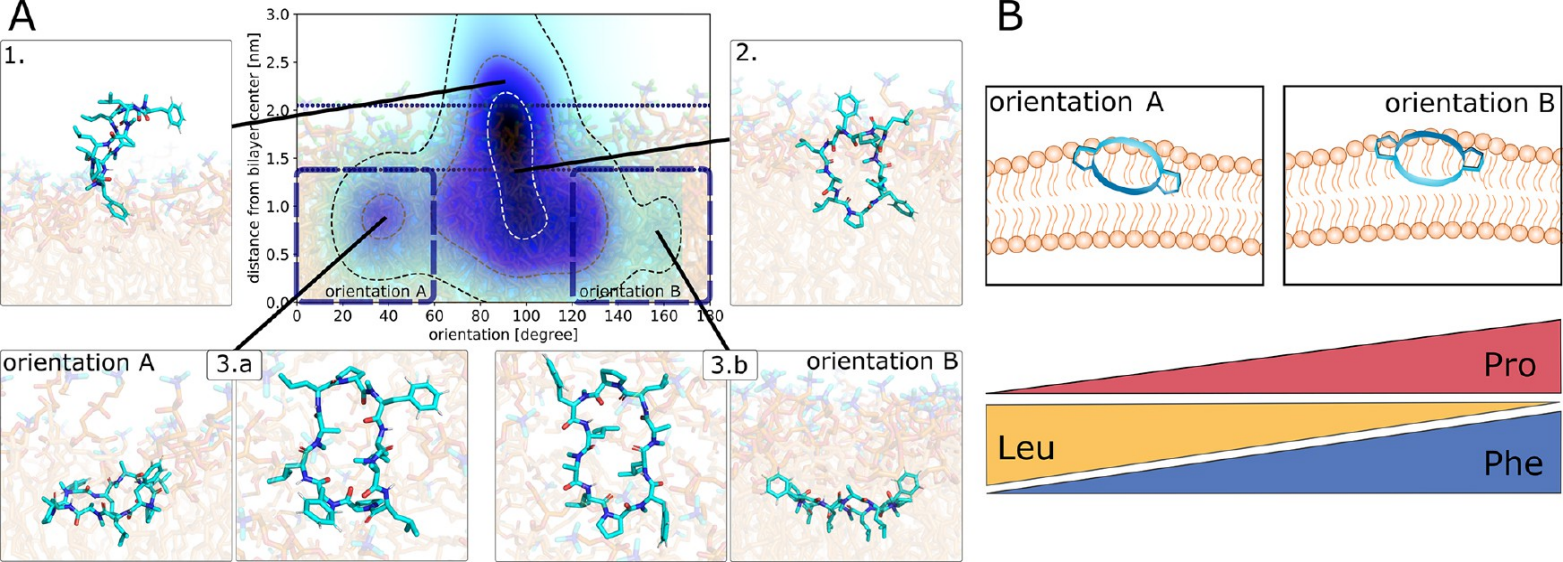
(A) Representation of how cyclic peptides insert into lipid membranes using the example of CDP **3**. The coordinates of the peptide are projected onto its distance from the bilayer center and its orientation with respect to the membrane plane. The heatmap shows the distribution of the simulation time spent in this phase space with darker color corresponding to more simulation time. Regions of interest are highlighted with simulation snapshots. The regions corresponding to orientation A and orientation B are marked with a dotted box. (B) Visualization of Table 2. The amino acid composition of the CDPs determines their preference for orientation A or B; proline and phenylalanine residues favor orientation B, and leucine residues favor orientation A.

From the biased simulations, we calculated the fraction of successful anchoring events for each potential anchor residue (Figure 4). For this, we pooled the pulling simulations of peptides that share the amino acid of interest. For example, CDP **1–7** all have a leucine at position 8. Thus, we combined the results of pulling at leucine 8 from these seven peptides. Note that this approach neglects how non-anchoring residues affect the anchor probability. However, as shown in Figures 3C and 5A.1, the non-anchoring side chains are distant from the interaction side and therefore unlikely to substantially impact anchoring. Interestingly, the anchoring pattern was very different when the peptides were in an open or in a closed state. In the closed conformation, the phenylalanine residues were the best anchors (Figure 4). All other residues showed only a very low probability. In contrast, the phenylalanine residues were the weakest anchors in open conformations, whereas the leucine residues at position 3 and 8 showed the highest probability. This difference can be explained by the different accessibility of the side chains in the two conformations. In the closed conformation, the leucine residues form a continuous hydrophobic patch and thus a single leucine is less accessible, while the phenylalanine residues are oriented outward.^69^ In open conformations, on the other hand, only leucine residues 1 and 6 form the continuous hydrophobic patch, while the leucines at positions 3 and 8 are positioned outward and accessible.^69^ In general, the overall anchor probability of the open conformations was higher. This is potentially due to the fact that open peptides can form stabilizing hydrogen bonds more easily and are more flexible. Thus, interactions between the apolar residues and the gaps created by thermal fluctuations in the lipids result more often in stable anchoring and subsequent insertion into the membrane.

### Effect of Membrane Composition on Headgroup Gaps

The probability of gaps to occur between the lipid headgroups is expected to affect the anchoring rate of the CDPs. To investigate how this probability is modulated by the addition of cholesterol molecules (as in biological membranes), we performed MD simulations of a pure POPC membrane and a POPC membrane with 30% cholesterol (without any CDP). The distribution of headgroup gaps was computed using Packmem.^83^ The details are shown in SI section 1.2.

In both cases, we observed an exponential decrease in headgroup gap probability with increasing gap size (SI Figure S1). Gaps up to an area of 0.60 nm^2^ and 0.40 nm^2^ were observed for pure POPC and POPC + cholesterol, respectively. The minimum gap size needed for a leucine side chain is approximately 0.23 nm^2^. Therefore, both lipid compositions lead to gaps that can accommodate amino acid side chains. However, very bulky anchor residues are likely not beneficial for the entry of the hydrophobic part of the membrane, although the stronger interactions formed by large anchors might compensate for this effect. We also find that headgroup gaps are less frequent in the membrane with cholesterol. This is expected as experimental results show that cholesterol decreases the flexibility of a membrane.^84^ Additionally, cholesterol is known to decrease membrane permeability.^85^ Nevertheless, headgroup gaps still occur and allow anchoring of the CDPs.

### Cyclic Peptides Occupy Two Distinct Orientations at Lipid Membranes

Starting from the anchored peptides, we performed elongated unbiased simulations to analyze how cyclic peptides behave inside lipid membranes. Figure 5A displays the entry pathway for a prototypical CDP (here for the example of open and closed states of CDP **3**). The plots for all CDPs are shown in the SI Figures S5 and S6. The coordinates of the peptide are projected to the distance of the peptide from the bilayer center (*y*-axis) and its orientation with respect to the membrane plane (*x*-axis). The blue heat map represents the density of simulations snapshots, i.e., the darker the color the more simulation points fall into that phase space. Note, however, that due to the rarity of the events we did not reach simulation equilibrium. Thus, the densities do not directly translate to free energies.

All peptides started anchored at the membrane (Figure 5A.1). In these starting structures, the COM of the peptides resided outside the membrane (distance to the membrane center >2.1 nm) and their longitudinal backbone axis was oriented nearly perpendicular to the membrane. The peptides stayed in this upright orientation while moving deeper into the membrane (Figure 5A.2), as it both minimizes the perturbation of the membrane and facilitates hydrogen bonding with the polar headgroups (Figure 3). Once the headgroup region was passed, the peptides started to rotate in one of two stable orientations. In both orientations, the peptides lay nearly parallel to the membrane plane at the interface between the headgroups and the tails (Figure 5A3.a,b). A comparison of the entry pathway of peptides in the closed and open conformations revealed that closed peptides penetrated deeper into the membrane (SI Figure S6) and only occupied one orientation, which we will term orientation B. Peptides in open conformations occupied both orientation A and B with a preference for orientation A. We also observed rotation from one orientation to the other. An example trajectory for such a rotation is shown in SI Figure S7.

**Figure 6:**
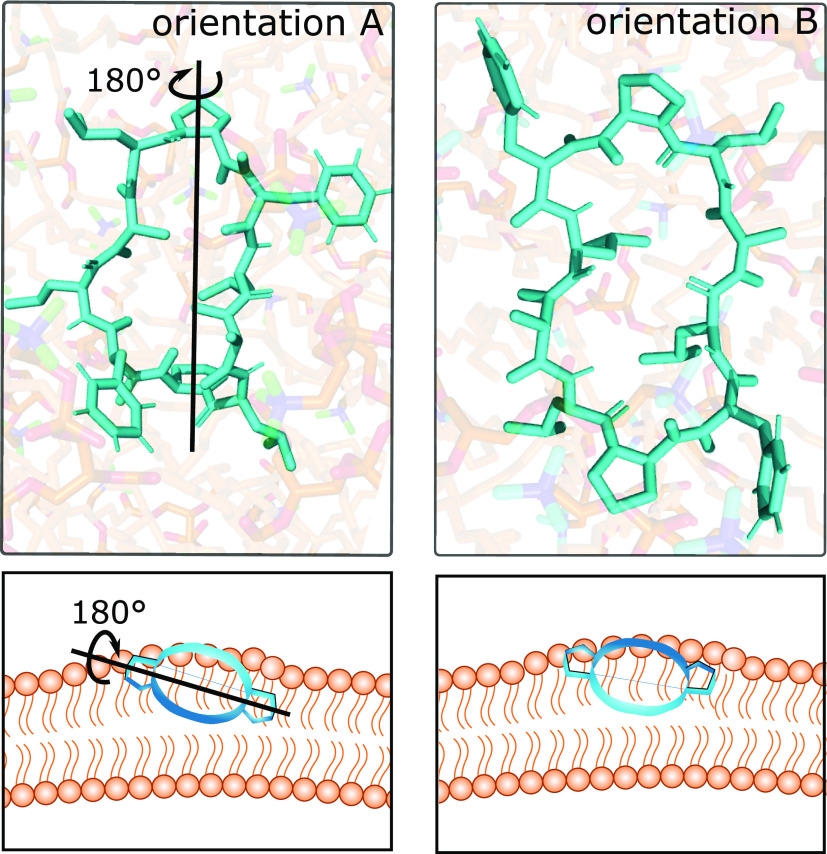
Representative snapshots of orientation A and B using the example of CDP **3**. The peptide is shown at the polar/apolar interface of the membrane and in a top view. To reach orientation B from orientation A, the peptide rotates by roughly 180° around its major axis. In addition, the ϕ-angles of leucine residues 3 and 8 show a ∼160° shift such that the leucine side chains are approximately aligned with the lipid tails. A cartoon was added for visual guidance.

The difference between orientation A and B is highlighted in Figure 6. The peptide is depicted in a top view at the membrane. In comparison to orientation A, orientation B is rotated roughly 180° along the major axis of the peptide backbone. In both orientations, the leucine residues approximately align with the lipid tails. To distinguish the two orientations in the cartoon representation, we chose to depict the proline residues. The proline ring is peaked toward the membrane middle in orientation A and toward the aqueous phase in orientation B.

At a first glance, it may be surprising that the CDPs orient parallel to the membrane, because this leads to a larger perturbed area in comparison to a perpendicular orientation. Interestingly, in our previous work, where we simulated CDPs at a water/chloroform interface, we observed the same two orientations and identified them as energetic minima (see SI Figure S8 for a comparison).^69^ We hypothesize that the parallel orientation is more favorable as the hydrophobicity profiles of the CDPs and the membrane match (see also panel 4 in Figure 1). These favorable interactions seem to outweigh the penalty of membrane perturbation. In addition, in both orientation A and B, the leucine residues align with the lipid tails, possibly reducing the entropic cost. Furthermore, the observed positioning of the CDPs at the interface of the polar headgroups and apolar tails is also in line with the results of previous studies using enhanced sampling simulations that located the free energy minimum in this region.^46^

### Amino Acid Composition Influences the Preferred Peptide Orientation

Previously, we found that the amino acid composition determines the orientation preference of CDPs at a water/chloroform interface.^69^ Therefore, we tested whether this finding was similar in the lipid bilayer system. Table 2 lists the relative fraction of simulation time spent in either orientation A or B for peptides in their open conformations.

**Table 2:** Relative Fraction [%] of Simulation Time Spent in Orientation A and B after Equilibration for CDPs **1**–**8** in Open Conformations

CDP	**1**	**2**	**3**	**4**	**5**	**6**	**7**	**8**
orientation A	87	95	71	89	96	69	53	33
orientation B	13	5	29	11	4	31	47	67

Encouragingly, the numbers in Table 2 match the fractions found in the water/chloroform system well (see SI Figure S9). With the only exception of CDP **8**, where the fraction differed by a factor of 2, the mean relative difference was only 8%. Three general trends can be observed in Table 2 and ref (69), which are summarized in Figure 5B. (1) The presence of proline in the peptide increases the fraction of orientation B. (2) The presence of phenylalanine also increases the fraction of orientation B. Here, we even observed a titratable effect. Peptides without phenylalanine in their sequence (e.g., CDP **5**) show the smallest fraction of orientation B, followed by peptides with one phenylalanine (e.g., CDP **4**). Peptides with two phenylalanines (e.g., CDP **3**) have the highest fraction of orientation B. (3) The presence of leucine increases the fraction of orientation A in a titratable manner (e.g., CDP **3** versus **6**/**7** versus **8**). Note that the percentages in Table 2 display the distribution after 100 ns simulation time. Thus, these numbers reflect the initial orientation distribution after membrane insertion. These clear sequence-specific differences in the orientation preferences of the peptides naturally raise the question of how the orientation influences the permeation process. Indeed, as we show below, the propensity of orientation B appears to be decisive for CDPs to interconvert to the closed conformation in the membrane, which is a necessary prerequisite to cross the lipid bilayer. Therefore, introducing structural modifications to favor orientation B might be a valuable consideration when designing permeable cyclic peptides.

### Open Cyclic Peptides Can Close Inside the Lipid Membrane

**Figure 7:**
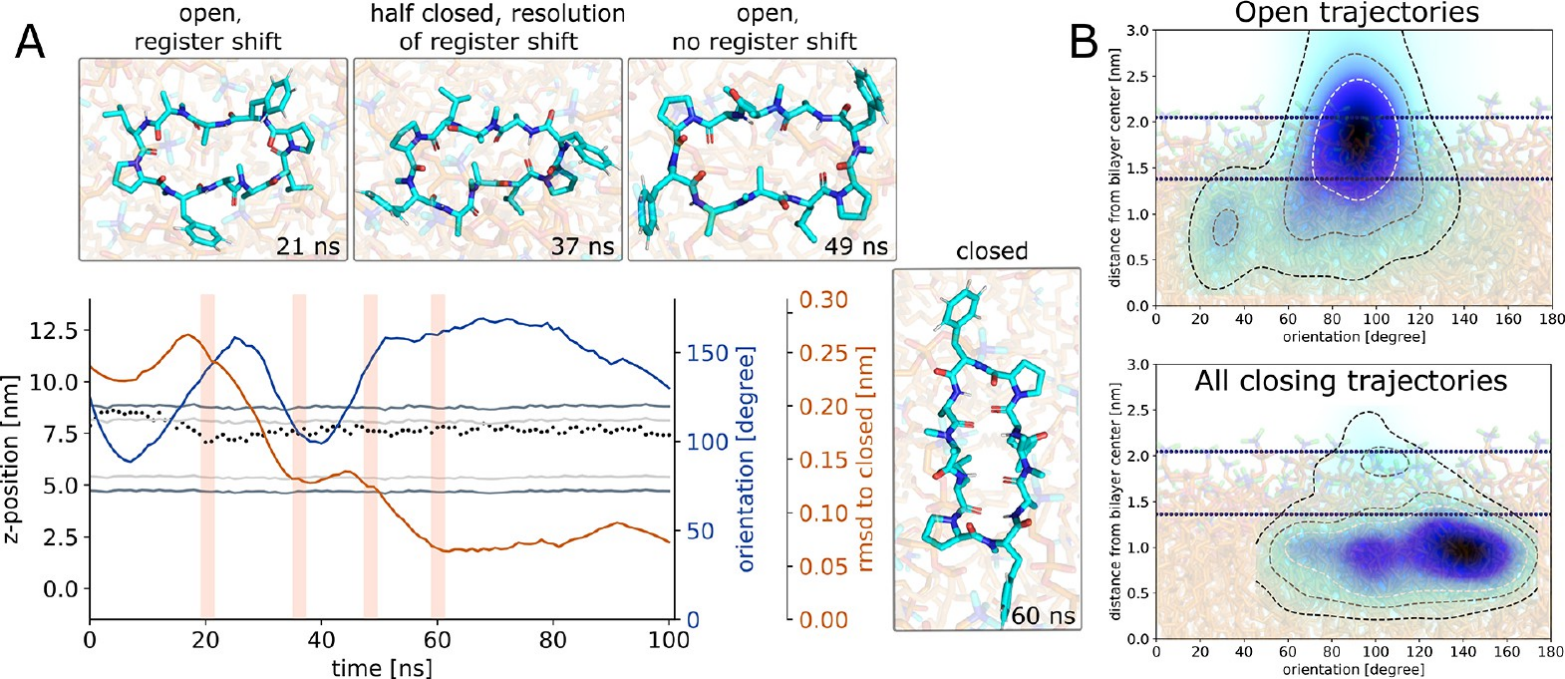
(A) Representative closing simulation using the example of CDP **6**. The peptide starts in an open conformation and closes inside the membrane via a half-closed structure. The closing of the peptide is traced by its RMSD with respect to the closed reference conformation (orange line). Inlays show selected simulation snapshots. Shaded areas correspond to the time point of the inlays. The dotted line indicates the *z*-position of the peptide. The membrane position is shown for reference (gray). The peptide stays in orientation B for the whole simulation (blue line). (B) Heatmap comparison of the orientation/position of all simulation frames in open conformations (here for CDP **6**) versus the orientation/position of the 12 closing trajectories. Whereas open peptides prefer orientation A, all open peptides that close during our simulations originate from orientation B.

In our simulations, we observed that CDPs can insert into lipid bilayers both in closed and in open conformations (see SI Figure S3). Interestingly, the CDPs retained their conformational flexibility inside the bilayer. Thus, also inside the membrane we observed multiple opening and closing events. Figure 7A displays a prototypic closing event within the membrane environment. The peptide enters the membrane in an open conformation (RMSD to the closed conformation of >0.2 nm). After the initial anchoring phase (until ∼18 ns), the peptide adopts orientation B, as indicated by its high orientation angle (until ∼30 ns). The displayed inlay at 21 ns also shows that the residues of the peptide are shifted by one position in comparison to their location in the closed structure, which we termed “register shift”. The closing is initiated by the formation of a first hydrogen bond on one side leading to a “half-closed” conformation (∼40 ns). In this half-closed conformation, the residues relocate their relative position, and the register shift is resolved. In order to fully close, the formed hydrogen bond is broken again, thus leading to an open conformation without a register shift. After further backbone torsional changes, the peptide is finally in the closed conformation (∼60 ns).

In total, we observed 13 closing events and 13 half-closing events across the different CDPs (see SI Table S1). Importantly, all closing and half-closing events started in orientation B. Although the peptides spent on average eight times more simulation time in orientation A, no closing event originating from orientation A was observed. Figure 7B illustrates this finding. The top panel shows the projected coordinates of all frames in open conformations of CDP **6**. This analysis already reveals the preference for orientation A when the CDP is in an open conformation. The bottom panel shows the projected coordinates of all frames in open conformations that subsequently close. They are exclusively found in orientation B. Furthermore, as described above, peptides that already enter the membrane in the closed conformation only occupy orientation B (see also SI Figure S6). In summary, these observations suggest a link between the orientation of the peptide in the membrane and its “internal” conformational preferences. While orientation A appears to prohibit rearrangements to the closed conformation, orientation B shifts the ensemble population toward it and closing events become possible within our simulation time. Thus, we argue that the phase-space overlap between the open peptides in orientation B and the closed peptides may facilitate the closing. These conclusions again match the central findings from our previous work at the water/chloroform interface.^69^ Open peptides that were in orientation B occupied a higher energy state and showed faster closing kinetics than peptides in orientation A. Additionally, Markov state models (MSMs) on that simulation data revealed that some CDPs with a low closed population in water had a significantly higher closed population at the interface. We hence hypothesize that the membrane has a similar “catalytic” effect on the conformational behavior of the CDPs and can facilitate a population shift toward the closed conformation. To test this hypothesis, we constructed a MSM for CDP **4** as it was the best sampled peptide in the membrane environment. The implied time scale plot of the MSM and a comparison between the MSM for CDP **4** at the water/chloroform interface and the membrane are shown in SI Figures S10 and S11. Both models identified a closed and a half-closed metastable state that only occupied orientation B. The open metastable states were split based on their orientation. Only orientation B showed transitions into the closed and half-closed conformation. More extensive studies on the other CDPs, potentially using enhanced sampling^86^ and dynamic reweighting,^87^ will be required to test the “catalytic” hypothesis further. However, from a methodological point of view, our combined works so far indicate that highly valuable mechanistic insights can be collected by approximating the membrane interface with a water/chloroform system. This is particularly interesting from a practical perspective as the latter system is significantly less computationally demanding.

Taken together our observations so far imply that cyclic peptides can cross membranes via two pathways: (1) In the “prefolding” pathway, peptides have a significant population of the closed conformation already in the aqueous solution. In this conformation, they are able to insert into the membrane and cross it. (2) In addition, our simulations suggest that cyclic peptides can enter the membrane in open conformations and close inside the membrane. Thus, we showed that also peptides without prefolding can possibly achieve passive permeability if closing is sufficiently favorable inside the membrane. This opens up a new realm of design considerations that focus on increasing the closed population at the lipid interface in addition to prefolding in water.

### Phenylalanine Residues Can Act as a “Lock” That Prevents Closing

**Figure 8:**
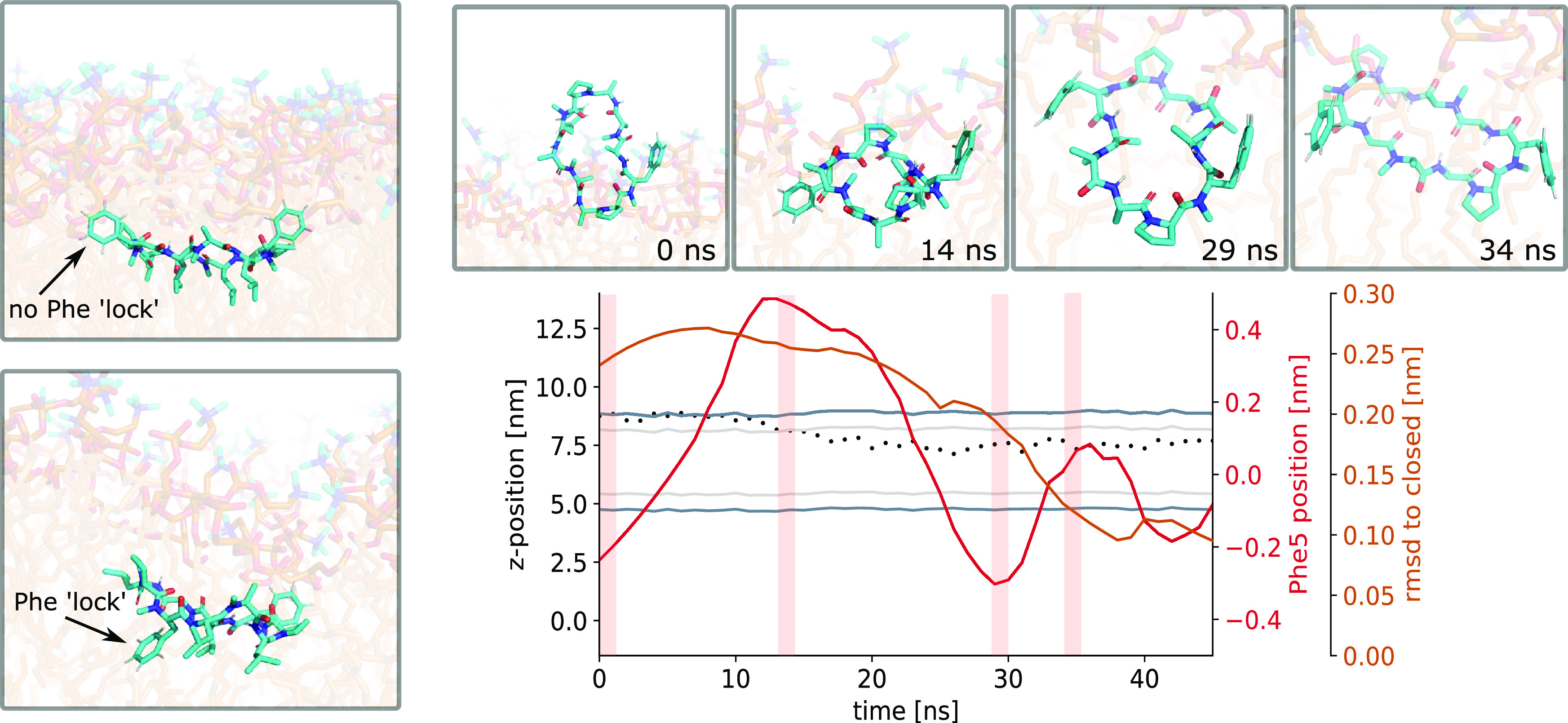
Phenylalanine can act as a lock that prevents closing in the membrane. (left) Phenylalanine can adopt two distinct positions in orientation B. In the unlocked position, both phenylalanine residues point toward the aqueous phase. In the “locked” position, at least one phenylalanine residue is rotated and points toward the membrane center. All closing events originate from the unlocked position. (right) Simulation of CDP **8** that shows an unlocking and a closing event. The closing of the peptide is traced by the RMSD with respect to the closed reference (orange line). The red line indicates the relative position of phenylalanine at position 5 with respect to the ring plane of the peptide. Inlays show selected simulation snapshots. Shaded areas correspond to the time point of the inlays. The dotted line indicates the *z*-position of the peptide. The membrane position is shown for reference (gray). The peptide first adopts orientation B in the locked position after entering the membrane. After a rotation of phenylalanine residue 5 to the unlocked position, the peptide starts closing.

We observed in our simulations that CDPs only closed when in orientation B. Although all closing events originated from orientation B, not all peptides in orientation B closed. Therefore, we investigated the difference between the closing and non-closing peptides in orientation B. We found that the residue position at the β-turn of the CDPs (positions 5 and 10, in this CDP series either phenylalanine or alanine) was decisive for closing. The left panel of Figure 8 illustrates this difference. In the top snapshot, the two phenylalanine side chains at the β-turn both point in the same direction toward the aqueous solution. In this position, the peptide backbone is able to interconvert to the closed conformation. In contrast, if the two residues point in different directions, this creates a “lock” that prevents the peptide from closing (bottom snapshot). The right panel of Figure 8 shows a simulation where the peptide enters the membrane in an open conformation and adopts orientation B. Initially, the two phenylalanine residues point in different directions (∼14 ns). In this specific example, phenylalanine at position 5 points toward the membrane center. Despite the bulkiness of the phenylalanine residue, it rotates away from the locked position until both phenylalanine residues face toward the aqueous phase (∼29 ns). Shortly after this shift (and thus the release of the lock), we observed the closing of the peptide. We validated the lock hypothesis using biased simulations (see SI Methods section 1.1). Starting from a locked position, we applied a small force to the dihedral angle of the phenylalanine backbone torsion that pulled it to the unlocked position. Indeed, as shown in SI Figure S12, this led to releases of the lock and subsequent closing events.

These findings highlight the contextual role of bulky residues like phenylalanine at position 5 and 10. On the one hand, they are the most efficient anchors in the closed conformation. On the other hand, their bulky nature can hinder conformational closure if they are in the locked position. Consequently, these observations emphasize again that for an optimal design strategy, it is crucial to know the prevalent permeability pathway of a cyclic peptide. Prefolded peptides enter the membrane in the closed conformation and thus rely on anchors at the β-turns. In addition, they do not need to close inside the membrane as they are already in the closed conformation. In contrast, peptides, which are mostly in open conformations in water, prefer anchors at different positions but have to close inside the membrane. For such peptides, it might be beneficial to have less bulky residues at the β-turns.

### Crossing From the Upper to the Lower Leaflet Requires Anchoring and Flipping

After entering the lipid membrane, the cyclic peptides have to cross from the upper leaflet to the lower leaflet in order to fully permeate through the membrane. Previous research has shown that this is associated with an energy barrier that can be higher than the barrier for entering the membrane.^46^ Indeed, also in our simulations, leaflet crossing was the rarest of the membrane permeation steps we observed. However, we were able to observe five permanent and three transient unbiased leaflet crossing events. Only peptides in the closed conformation penetrated deep enough into the bilayer to lose all water interactions (see SI Figure S13) and subsequently cross to the lower leaflet. This is in line with our observation that only closed peptides fully enter the apolar phase in a polar/apolar interface system.^69^

**Figure 9:**
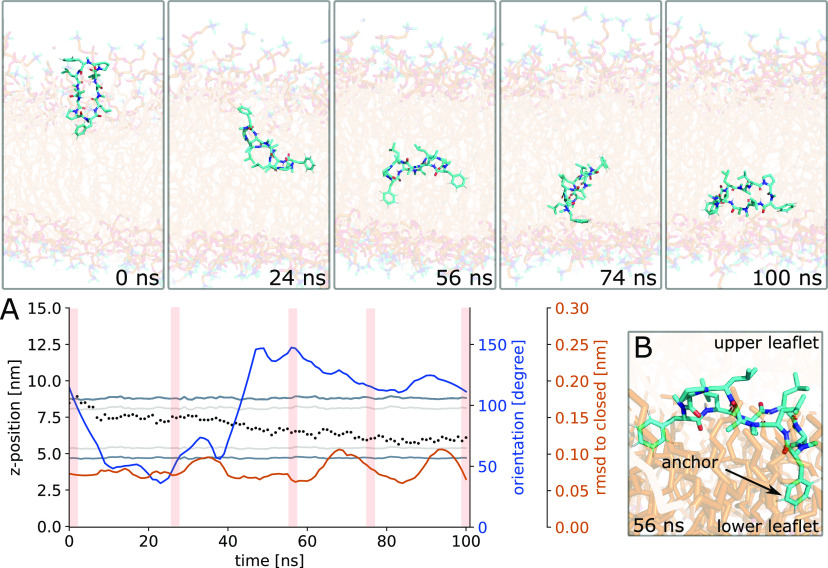
(A) CDP **3** crossing from the upper to the lower leaflet. Inlays show representative simulation snapshots. Shaded areas correspond to the time point of the inlays. Upon passing the membrane center, the peptide undergoes a flip along its major axis (blue line). (B) Zoom-in on the peptide anchoring in the lower leaflet. Lipid tails from the upper and lower leaflet are colored differently to help distinguishing the two leaflets.

Figure 9A and the movie in the SI show an unbiased leaflet crossing event for CDP **3**. The closed peptide first enters the membrane in the typical upright position (0–5 ns). After penetrating deeper into the lipid membrane, it adopts orientation B, which is the stable orientation for closed peptides (24 ns). In this orientation, the proline residues point toward the upper aqueous phase and the leucine residues align with the lipid tails. Most of our simulations remain in this state for the entire course of the simulation. However, we also observed some rare leaflet crossing events that were associated with a flip of the peptide as shown in Figure 9 at 56 ns. During the movement into the lower leaflet, the peptide rotates roughly 180° along its major axis. Figure 9 shows a fast flipping event where the peptide performed the full flip within a few nanoseconds, while other simulations also showed a slower and more gradual flipping.

A closer inspection of the flipping process revealed two underlying principles. First, the flip was triggered by another anchoring event. Anchoring of one of the peptide residues in the lipid tails of the lower leaflet preceded the flip and leaflet crossing. Figure 9B shows a zoom-in on this anchoring. Here, the lipid tails of the upper and lower leaflet are colored differently to aid the visual inspection. Using the knowledge of the initial anchoring events, we reseeded simulations from anchored positions and obtained 18 additional unbiased leaflet crossing events. Second, our orientation analysis had shown that closed peptides exclusively occupied orientation B (see SI Figure S6). In orientation B, the leucine residues point toward the membrane center and the phenylalanine and proline residues point toward the aqueous phase. In order to adopt this orientation in both leaflets, the peptide has to rotate 180° while passing through the membrane center. Thus, flipping is necessary to adopt the more favorable orientation B for closed peptides at the lower leaflet. Indeed, in the three transient leaflet crossing events we observed no flipping, and thus the peptides diffused back to the original leaflet after a few nanoseconds. Flipping along its major axis requires larger motions of the peptide in the rather viscous and sterically hindered lipid-tail environment. We reason that the anchoring and the orientation change go hand in hand to overcome the leaflet crossing barrier.

**Figure 10:**
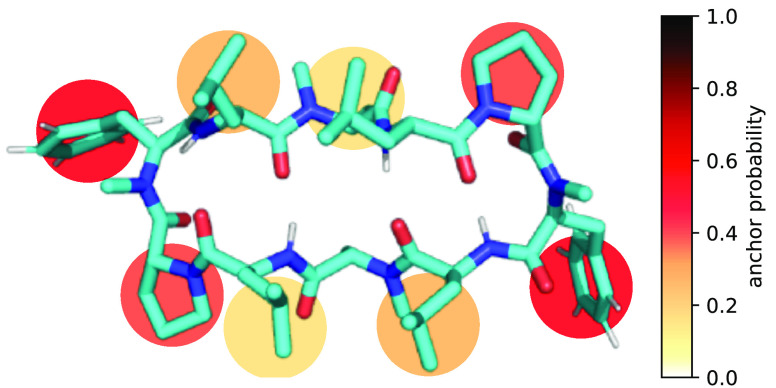
Ability of the different side chains of the CDPs to anchor in the lower membrane leaflet when the peptide is inserted in the upper leaflet. Membrane crossings were observed only for peptides in the closed conformation. The anchor probability was determined as the fraction of successful anchoring events after pulling the CDPs on that respective side chain toward the lower leaflet.

To assess the anchor quality of the different amino acid residues for leaflet crossing, we again performed pulling simulations as described above. Using these simulations, we were able to increase the rate of leaflet crossing events by a factor of 140. The resulting probabilities are depicted in Figure 10. The results resemble the anchor probabilities for entering the membrane in the closed state (Figure 4) with the difference that the total values are higher for the leaflet crossing event. The phenylalanine residue is again the best anchor. As the local environment in the upper and lower leaflet are identical, there is no environmental change associated with the anchoring. Thus, the different anchor probabilities can be attributed to differences in accessibility, with the phenylalanine residue being the most exposed in the closed conformation.

### Four Steps of Membrane Permeation

Figure 1 summarizes the four steps of passive membrane permeation that we identified for the series of CDPs. We found that the first step involves the anchoring of an exposed residue to a transient gap between the lipid headgroups (Figure 3). This process is stabilized by hydrogen bonds between the backbone atoms of the peptide and the headgroups. Interestingly, using machine learning techniques on a large compound library, Rzepiela et al.^6^ found that an asymmetric accumulation of hydrophobicity on one side of a compound is predictive for highly permeable macrocycles. The authors presume that hydrophobic regions might enter the membrane first and thus catalyze the entry of the remaining molecule. This hypothesis is in line with the mechanistic insights gained from our simulations, where the apolar residues act as anchors and enter the membrane first. The CDPs could insert into the membrane in both open and closed conformations, with different residues as main anchors. Interestingly, open conformations showed a higher total probability of entering the membrane due to an increased ability to form stabilizing hydrogen bonds with the lipid headgroups.

The second step is insertion and orientation. In the bilayer, the peptides locate themselves at the interface between the apolar tails and the polar headgroups. While peptides in open conformations can adopt two different orientations, A and B, that differ by a 180° rotation (Figure 6), closed peptides occur only in orientation B. In line with our previous work, we found that the amino acid composition modulates the fraction between orientation A and B for the open peptides (Figure 5 and Table 2). If the peptide entered the membrane in an open conformation, it has to interconvert to the closed conformation to be able to cross the membrane core. Importantly, only open peptides in orientation B interconvert to the closed conformation (Figure 7). Given the importance of orientation B for closing, optimizing CDPs to occupy orientation B might be of interest in designing peptides. In our simulations, both the presence of proline and phenylalanine as well as the absence of leucine enhanced the fraction of orientation B. However, both the phenylalanine and leucine residues have an ambivalent role. As shown in Figure 8, phenylalanine residues can also act as a lock and prevent closing. Leucines favor orientation A but are also important anchors for peptides in open conformations.

The fourth step is the crossing from the upper to the lower leaflet. We found that this process again involves anchoring of an exposed residue—this time to the lipid tails of the lower leaflet. In addition, the peptide flips when passing the membrane center (Figure 9). Thus, in each leaflet the CDP adopts the favorable orientation B within the membrane. Given the major lipid rearrangements necessary to allow the flip of molecules as large as the CDPs in this study, bulky amino acids are expected to reduce the flipping rate.

Taking into account all studied permeation steps and the various—and sometimes conflicting—roles amino acids play in those steps, it is not surprising that the effects of the amino acid composition are highly contextual. Although the data set is small, we can make some observations.

#### Leucines

Leucine residues are important anchors for open peptides but favor orientation A inside the membrane and are relatively bulky. Starting from CDP **3** and removing half of the leucine residues leads to CDPs **6** and **7**, which have an 8–15 fold increased permeability over CDP **3** (Table 1). The remaining two leucine residues appear to be sufficient to ensure reasonable anchoring rates while the probability of orientation B is significantly increased (Table 2). However, if all leucine residues are removed as in CDP **8**, the permeability is decreased by a factor of 12 compared to CDP **3**, even though the fraction of orientation B is even higher (Table 2).

#### Phenylalanines

Phenylalanine residues showed a special significance throughout all four steps of permeation. For peptides in the closed conformation, they showed the highest anchor probability for entering the membrane as well as for crossing to the lower leaflet. For peptides inserting in open conformations, the bulkiness of phenylalanine hinders the interconversion to the closed permeable conformation (lock). In water, CDP **1** is found almost exclusively in open conformations, whereas CDP **3** has a significant population of the closed conformation (<1% versus 45% closed fraction, respectively).^67^ Thus, the replacement of all phenylalanine residues affects these two peptides differently. The open CDP **1** does not rely on phenylalanine to anchor to the membrane but benefits from the removal of the locks. Thus, we observe a large 80-fold increase in permeability upon replacement of all phenylalanine residues resulting in CDP **2** (Table 1). In contrast, replacing all phenylalanine residues of CDP **3** leads to CDP **5**, which is only associated with a moderate 5-fold permeability increase (Table 1).

**Figure 11:**
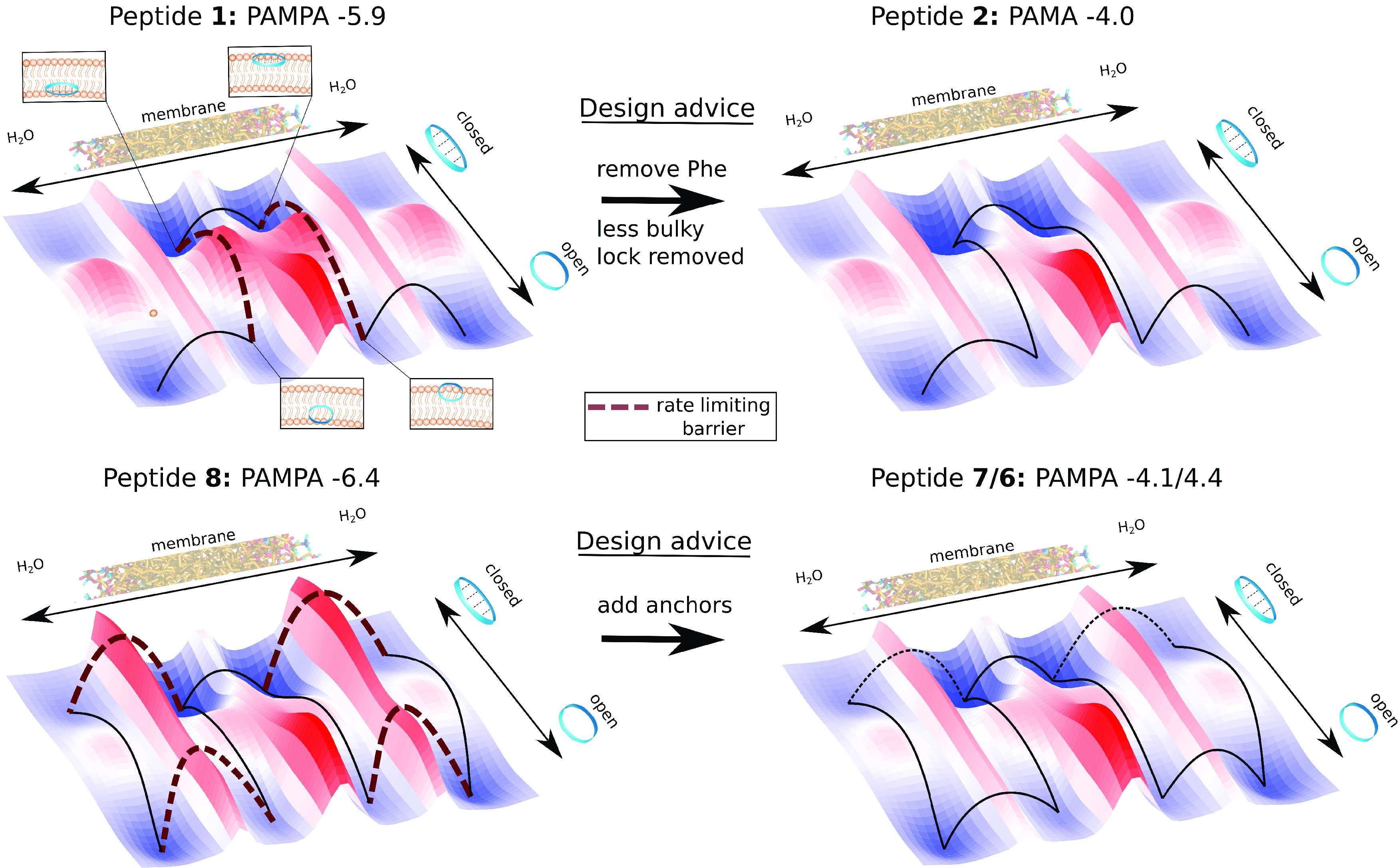
Schematic free-energy surfaces and the corresponding rate-limiting barriers for the impermeable CDPs **1** and **8** (conformational closure for CDP **1** and anchoring for CDP **8**). The free energies are projected to the conformation of the peptide and its position with respect to the membrane. Design advice for lowering barriers and thus improving the passive permeability are indicated.

Based on these observations, we can draft schematic free-energy surfaces for the permeation process of the impermeable CDPs **1** and **8** and how it may change upon modifications (Figure 11). Note that these are hypotheses. The main energy barriers are associated with the interconvertion between the closed and open states in water and in the membrane, passing through the lipid headgroup region, and leaflet crossing. As shown in our previous studies^67,69^ and based on the fact that CDP **1** was the only peptide where not a single closing or half-closing was observed (see SI Table S1), CDP **1** suffers from both an unfavorable ratio between open/closed states and high interconversion barriers. To improve interconversion within the membrane, our permeation model suggests to remove the bulky phenylalanine residue(s) that can lock the peptide in open conformations inside the membrane, leading to CDP **2** which has a significantly higher permeability. For CDP **8**, in contrast, we observed a high interconversion dynamic with multiple closing and opening events (SI Table S1). However, CDP **8** contains no leucine residues. In our permeation model, this should decrease its anchoring potential, especially in open conformations. Indeed, in this study not a single anchoring event was observed for CDP **8** (see SI Table S1). This highlights that different energy barriers can become rate limiting for different cyclic peptides. In this case, introducing residues with good anchoring ability is advised by the model, leading to CDPs **6** and **7** with dramatically improved permeabilities.

## Conclusions

In this study, we investigated the pathway and main steps for the passive membrane diffusion of conformationally flexible cyclic peptides. Based on extensive simulations, we identified four steps of the membrane permeation process of cyclic peptides: (1) anchoring with residues in transient gaps between lipid headgroups, (2) insertion in the membrane and orienting parallel to the membrane plane in orientation B, (3) if the peptide enters in an open conformation, interconversion to the closed permeable conformation, and (4) leaflet crossing involving anchoring and rotation.

For the first step, the pulling simulations revealed that the main anchoring residues differ for open and closed conformations, due to the different accessibility of these residues in the two conformations. Given that the anchoring probability of amino acids is conformation dependent, characterizing the conformational behavior of cyclic peptides is therefore crucial for rational design. Knowing the predominant conformation of the peptide before entering the membrane and the exposed residues may help to optimize the amino acid composition for membrane permeable CDPs. Considering the anchor potential of residues for permeability is a new design concept that might be transferable to other compounds as well. A recent study by Morstein et al.^88^ identified medium-chain lipid conjugation as a general modulator of cell membrane permeability. Investigations of whether these medium-length lipid chains act as “anchors” will be part of future research.

In the second step, the peptides insert in the membrane and orient themselves parallel to the membrane plane. If the insertion occurs in an open conformation, two orientations A and B are possible. If the peptides insert already in the closed conformation (prefolding), only orientation B was observed. This design consideration should be applicable to other macrocycles with asymmetrically oriented large and continuous hydrophobic surface patches. Only orientation B is favorable for subsequent interconversion to the closed permeable conformation (step 3). We expect this to apply to chameleonic cyclic peptides in general.

For full permeation, the peptides have to cross the membrane center to reach the lower leaflet (step 4). An anchoring mechanism similar to the initial step was observed in connection with a rotation of the peptide to reach orientation B in the lower leaflet. Again, we reason that this step should be applicable to other macrocycles with asymmetrically oriented large and continuous hydrophobic surface patches.

The simulations show that the effect of amino acids (e.g., leucines, phenylalanines, prolines) can be highly contextual, i.e., it depends on the location of the amino acid in the peptide and on the other residues. The observations and hypotheses presented in this study are based on a small data set and limited influence parameters but nevertheless provide important insights and a highly detailed mechanistic model of the membrane permeation process of flexible cyclic peptides. While biasing was applied in the initial peptide–membrane association with a pulling force, all mechanistic conclusions were drawn from unbiased MD simulations. Previous simulation-based studies have either focused on significantly smaller and less flexible molecules or fully relied on biased simulations. Most importantly, our findings emphasize that solid understanding of the preferred conformation(s) of a peptide in solution may be decisive for the success of a lead optimization campaign targeting permeability. With the here provided atomistic and dynamic insights into the permeation pathway of cyclic peptides, we aim to inspire and stimulate new design principles and predictive modeling approaches for bioavailable macrocyclic drugs.

## Methods

### MD Simulations

The simulations were carried out with the Groningen Machine for Chemical Simulations (GROMACS) 2020.5 software package^89^ in combination with the GROMOS 54A8 force field^90^ and the POPC model of Marzuoli et al.^91^ Simulations were performed under periodic boundary conditions with a leapfrog integration scheme^92^ and a time step of 2 fs. Peptides, lipids, and solvent were coupled to three separate thermostats at 303 K using a weak coupling scheme^93^ and a relaxation time of 0.1 ps. A semianisotropic Parrinello–Rahman barostat^94^ at 1.0 bar with a coupling constant of 2.0 ps and isothermal compressibility of 0.45 nm^2^/N was used. Long-range electrostatics were treated using the particle mesh Ewald algorithm.^95^ Bond lengths were constrained using the linear constraint solver (LINCS) algorithm.^96^ A center-of-mass (COM) motion removal was applied every step to eliminate the movement of the bilayer relative to the solvent.

In order to avoid finite size effects, a relatively large lipid patch containing 512 POPC molecules was used. The CDP starting structures in the closed conformation were obtained by NMR spectroscopy.^81^ The protocol to obtain the starting structures in the major open conformation has been described in detail in refs (53, 67, and 69). The CDPs were initially positioned in the aqueous phase approximately 3 nm away from the headgroup region of the POPC bilayer. The system was solvated with the SPC^97^ water model and equilibrated using a 100 ps NVT thermalization and 1 ns NPT equilibration. Unbiased production runs (NPT) had a length of 100 ns.

### Biased Simulations

Pulling simulations were performed using the GROMACS internal pull code. Peptides started in the aqueous phase approximately 3 nm away from the headgroup region of the POPC bilayer. A constant pulling force of 50 kJ nm^–1^ mol^–1^ was applied on the distance between the COM of the selected anchor amino acid and the COM of the POPC lipids. The pulling force was chosen small enough so that, if applied on the COM of the peptide, it did not perturb the membrane or lead to a membrane insertion event (see SI Figure S4). To increase anchoring events, five pulling simulations with a length of 5 ns each were performed for every leucine, phenylalanine, and proline residue of the respective CDP. For the pulling simulations of leaflet crossing, 20 simulations with a length of 10 ns each were performed for every leucine, phenylalanine, and proline residue of the respective CDP.

### Data Analysis

If not stated otherwise, all trajectories were analyzed using the Python library MDTraj.^98^

#### Position

The *z*-coordinate of the COM was used to indicate the peptide position. As a reference, the headgroup position and the start of the tails of the POPC bilayer are shown in the figures. The average *z*-coordinate of the headgroups was calculated using the nitrogen position of the choline group. The start of the tails was calculated using the carbon atom succeeding the ester group.

#### Orientation with Respect to the Membrane

The peptide normal vector *a⃗* was determined using the cross product of the major and minor axis of the ellipse formed by the peptide backbone. As the membrane position remains approximately fixed throughout the simulation, the *z*-axis *b⃗* was used as an approximation for the normal axis of the membrane. The angle between these normal vectors was calculated as
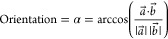
1

#### RMSD

The atom-positional backbone root-mean square deviation (RMSD) with respect to the NMR solution structure of the closed conformation was calculated using the Python library MDTraj.^98^

#### Treating Periodic Boundary Conditions

Due to the periodic boundary conditions, peptides can access the membrane from above and below. For consistency and didactic reasons, all trajectories were transformed such that the peptide entered the membrane at the upper leaflet. Thus, both the position and the orientation were shifted by a 180° rotation of the simulation box around the *x*-axis if the peptide entered from below.

## Data Availability

An example Jupyter notebook to analyze the CDP trajectories is available on GitHub (https://github.com/rinikerlab/decapeptides-membrane). This repository also contains the topology and structure files of the POPC system as well as topology files of the CDPs and the open and closed starting structures used in this publication. Further information, custom scripts, or production trajectories are available from the corresponding author (S.R.) upon request. The freely available software can be obtained via the following links: GROMACS (https://www.gromacs.org/) and PyMol (https://github.com/schrodinger/pymol-open-source).

## Supporting Information Available

Additional methods, Figures S1–S13, and Table S1 (PDF)
Movie of an unbiased anchoring event (MPG)
Movie of an unbiased leaflet crossing (MP4)
Movie of an unbiased lock removal event (MPG)

## Supplementary Material

jm2c01837_si_001.pdf

jm2c01837_si_002.mpg

jm2c01837_si_003.mp4

jm2c01837_si_004.mpg

## Abbreviations Used

CDP: cyclic decapeptide
MD: molecular dynamics
PAMPA: parallel artificial membrane permeation assay
POPC: 1-palmitoyl-2-oleoylphosphatidylcholine
RMSD: root-mean-square deviation
