# Supplementary material for: Lessons for Oral Bioavailability: How Conformationally Flexible Cyclic Peptides Enter and Cross Lipid Membranes
Source: J Med Chem. 2023 Feb 10;66(4):2773–88. doi: 10.1021/acs.jmedchem.2c01837 (PMC9969412; doi:10.1021/acs.jmedchem.2c01837)
Supplement: Supplementary file 1 — jm2c01837_si_001.pdf [file jm2c01837_si_001.pdf]

SUPPORTING INFORMATION
Lessons for Oral Bioavailability: How Conformationally Flexible Cyclic Peptides Enter and Cross Lipid Membranes

Stephanie M. Linker,[a‡] Christian Schellhaas,[a‡] Anna S. Kamenik,[a] Mac M. Veldhuizen,[a] Franz Waibl,[a] Hans-Jörg Roth,[b] Marianne Fouché,[b] Stephane Rodde,[b] and Sereina Riniker*[a]

[a] *Department of Chemistry and Applied Biosciences, ETH Zürich, Vladimir-Prelog-Weg 2, 8093 Zürich, Switzerland. E-mail: sriniker@ethz.ch*

[b] *Novartis Institutes for BioMedical Research, Novartis Pharma AG, Novartis Campus, 4056 Basel, Switzerland*

Contents

1 Additional Methods **S2**

1.1 Biasing Simulations for the Phenylalanine 'Lock' S2

1.2 Distribution of Head-Group Gap Size S2

1.3 Construction of Markov State Models S2

2 Additional Figures **S3**

3 Additional Tables **S16**

1 Additional Methods

1.1 Biasing Simulations for the Phenylalanine 'Lock'

Pulling simulations were performed using the GROMACS internal pull code for dihedral angles. An umbrella pulling force with a pulling rate of 0.1 nm and a force constant of 500 kJ nm^{-1} mol^{-1} was applied on the ψ backbone torsional angle of the phenylalanine. In total, 40 pulling simulations with a length of 20 ns were performed. This resulted in two successful 'lock removal' events.

1.2 Distribution of Headgroup Gap Size

The hydrophobic residues anchor to the membrane via gaps between the lipid headgroups. To better understand this process, we simulated a pure POPC membrane as well as a membrane containing a molar fraction of 30 % cholesterol. Consistently with the rest of this work, each membrane contained 512 POPC molecules. The second system contained additional 216 cholesterol molecules, which resembles the 30% cholesterol fraction observed in mammalian cells. This resulted in a 163.5 nm^2 sized membrane patch for pure POPC and a 169.5 nm^2 sized membrane patch for POPC+cholesterol (both after equilibration.) After an equilibration phase of 50 ns, we simulated each membrane for 150 ns, saving frames every 100 ps, and computed the occurrence of headgroup gaps. As the average lifespan of a gap is below 20 ps [1], we expect only weak correlations between consecutive frames.

We used Packmem [1] to analyze the occurrence of gaps. Packmem creates a 2D grid of 0.1 nm resolution along the x/y-coordinates of a leaflet of the membrane. At each chosen x/y-coordinate, the highest z-coordinate is determined, which overlaps with the van der Waals radius of a membrane atom. If this atom belongs to the hydrophobic part of POPC or chlosterol, the respective grid point is defined to be in a headgroup gap. The hydrophobic part of POPC was defined as all atoms below the carbon bound to the phosphate group, and that of cholesterol as everything but the C-OH group.

The area of a headgroup gap was computed by searching continuous patches of tiles that are in a gap. The occurrence of headgroup gaps of each size was normalized by the number of frames times two, because there are two leaflets. This gives us the probability of finding a headgroup gap of a given size in a single MD snapshot.

The probability distribution is shown in Figure S1. As a guide for the eye, we also show a rough estimate of the area needed for a leucine side chain to enter the membrane (0.23 nm^2). This number was computed by noting that the broadest part of the sidechain are the two methyl groups in front, and by modelling their area as the obround shape defined by two circles of 0.2 nm radius and a distance of 0.25 nm. We performed an exponential fit of the data to determine the decay constant for both membrane setups. As recommended in Ref. [1], we only considered headgroup gaps > 0.15 nm^2 for the fit. The decay constants were 0.06 nm^2 for pure POPC and 0.05 nm^2 for POPC+cholesterol.

1.3 Construction of Markov State Models

Markov state models (MSMs) allow for the calculation of equilibrium quantities and long-time kinetics from ensembles of short simulations [2]. MSM require 'local equilibrium' within the MSM states, but not a 'global equilibrium' between all MSM states.

The PyEMMA Python library [3] was used to construct the MSMs. The sine and cosine of the ϕ and ψ backbone torsional angles as well as the position with respect to the membrane center and the orientation of CDP **4** with respect to the membrane normal were extracted from the trajectories. This resulted in 42 input features, which were reduced to 12 collective coordinates by time-lagged independent component

analysis (TICA)[4]. The trajectory was clustered using k-means clustering with 30 states. A lagtime τ of 10 ns was chosen to ensure Markovianity. To group the microstates into metastable conformational states, robust Perron cluster-cluster analysis (PCCA+) [5] was performed.

2 Additional Figures

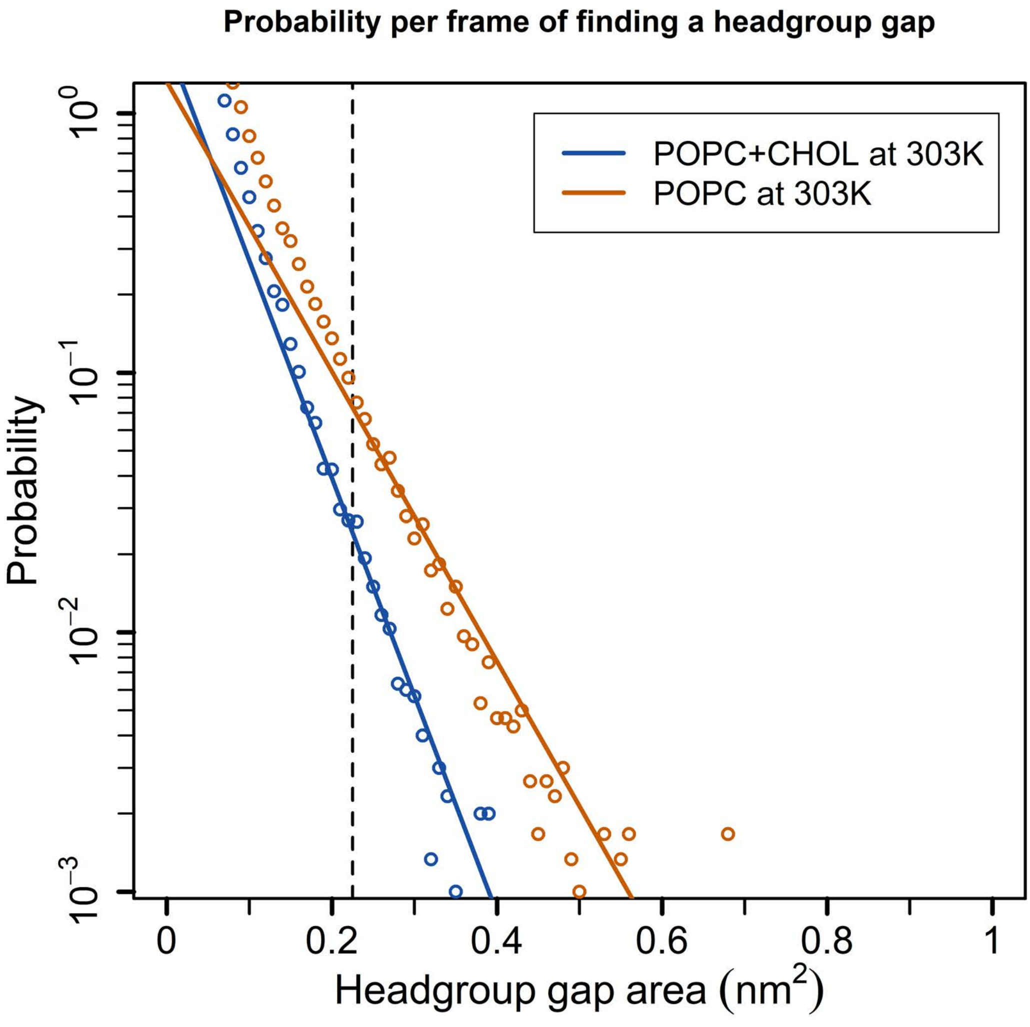

Figure S1: Probability of finding a headgroup gap of a given size in a pure POPC membrane (orange) or a POPC membrane with 30% cholesterol (blue) at 303 K. The straight lines show a linear fit on the logarithmic probability values, omitting points below a probability of 1/1000 and below an area of 0.15 nm^2. The dashed line is at 0.23 nm^2, as an estimate of the area required by a leucine sidechain (as explained above).

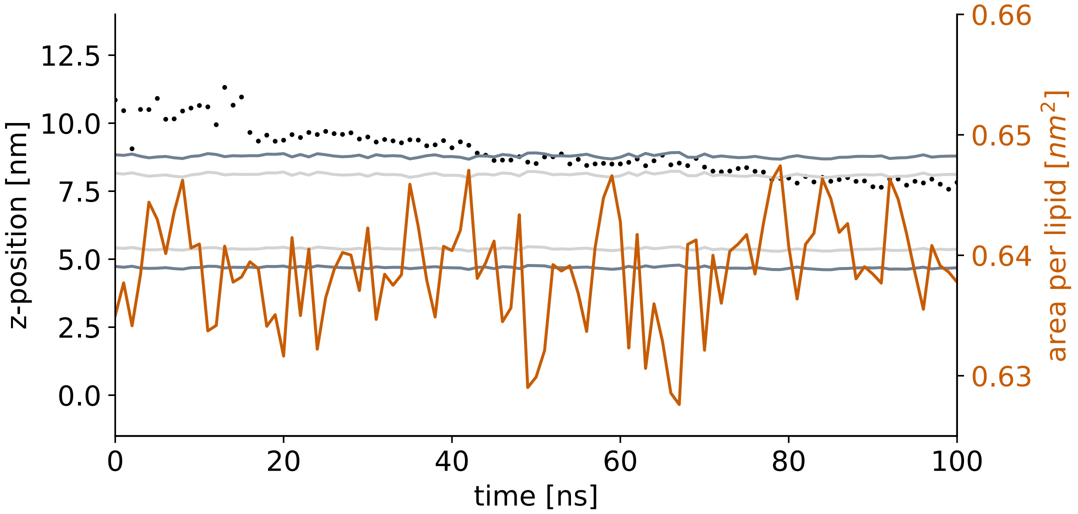

Figure S2: Change in the area per lipid (brown, right axis) over time. The z-position of the CDP **1** is indicated with black dots. The position of the headgroup region and lipid tails are indicated with dark grey and light grey lines, respectively. No significant changes in the area per lipid were observed upon the peptide entering the membrane (starting at 23 ns).

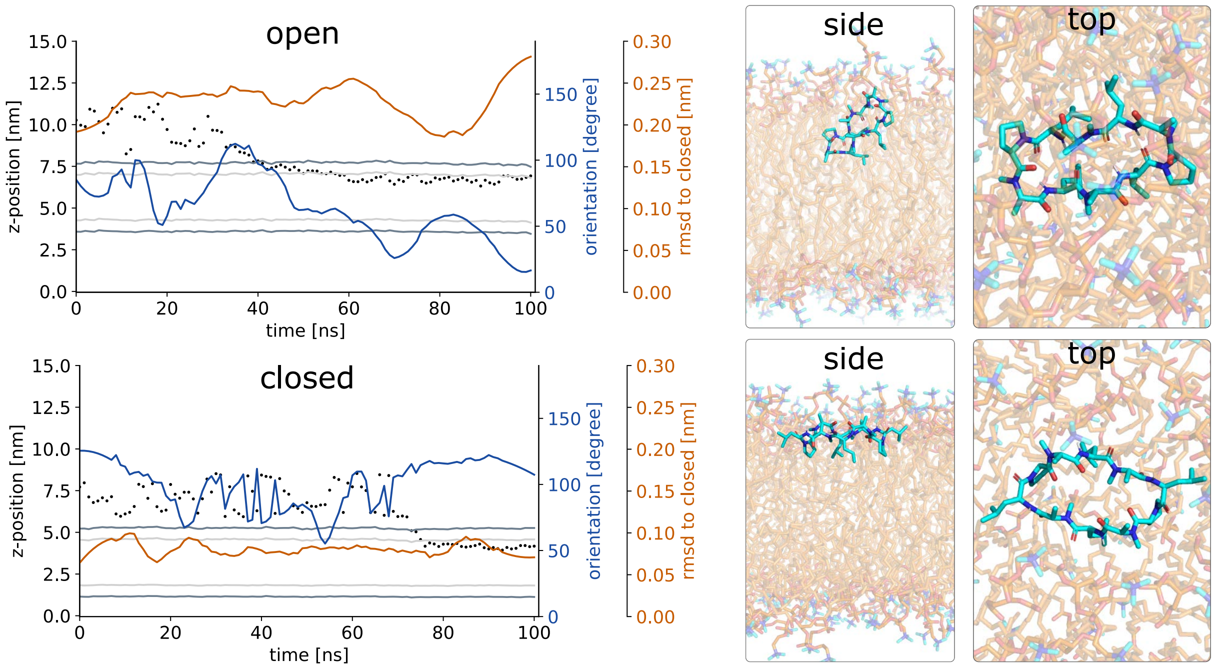

Figure S3: (Left): Trajectory of CDP **5** entering the membrane in the closed and in the open conformation. The z-position of the CDP is indicated with black dots. The position of the headgroup region and lipid tails are indicated with dark grey and light grey lines, respectively. The angle between the normal vectors of the peptide and the membrane is shown in blue. The RMSD with respect to the 'closed' conformation of the CDP is shown in orange. (Right): Snapshots of the CDP at the end of the simulations. In both cases, the peptide backbone is nearly parallel to the membrane plane.

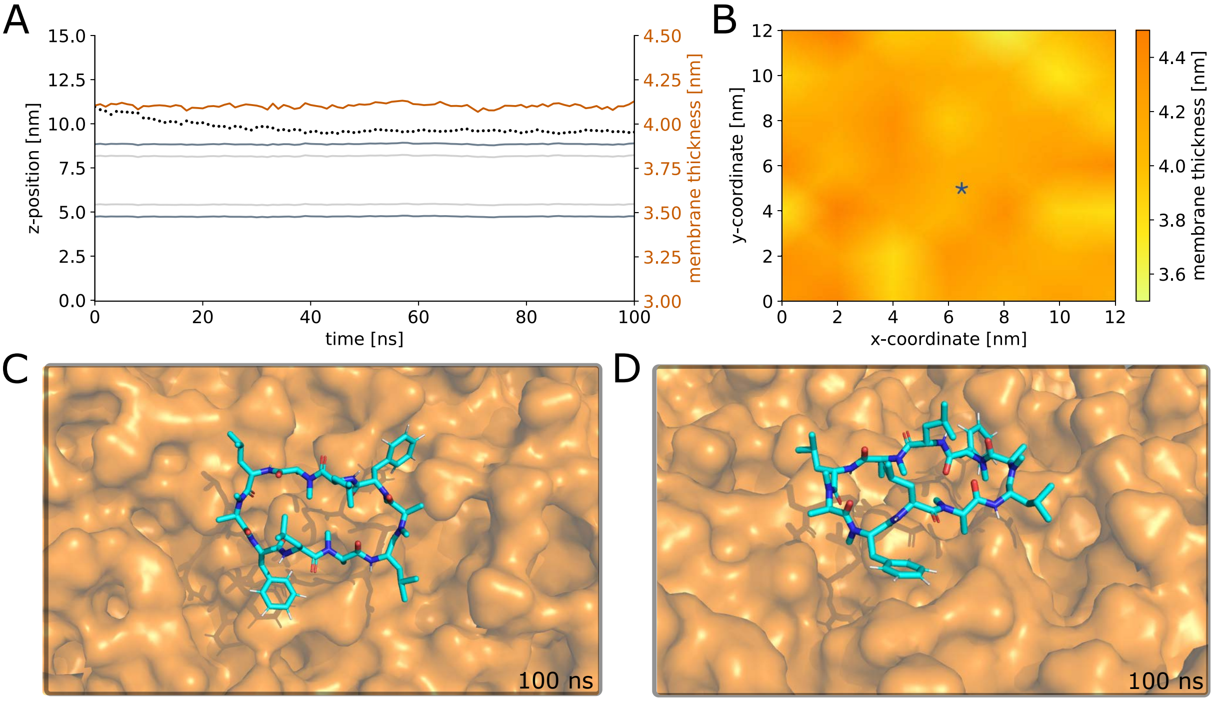

Figure S4: Pulling on the center-of-mass (COM) does not lead to membrane insertion or membrane crossing. A COM pulling force identical to the anchor pulling force (see Method section) was applied to the peptide. (**A**): The peptide is pulled to close proximity of the membrane but does not enter. The average membrane thickness over time is shown in orange. (**B**): Heatmap of the membrane thickness for simulation frame 100 ns. The position of the peptide is indicated with a blue asterisk. No substantial fluctuations in membrane thickness were observed around the peptide. (**C**): Top view of the peptide with COM pulling towards the membrane at frame 100 ns. The membrane surface is shown in orange. (**D**): Same as **C** but in side view.

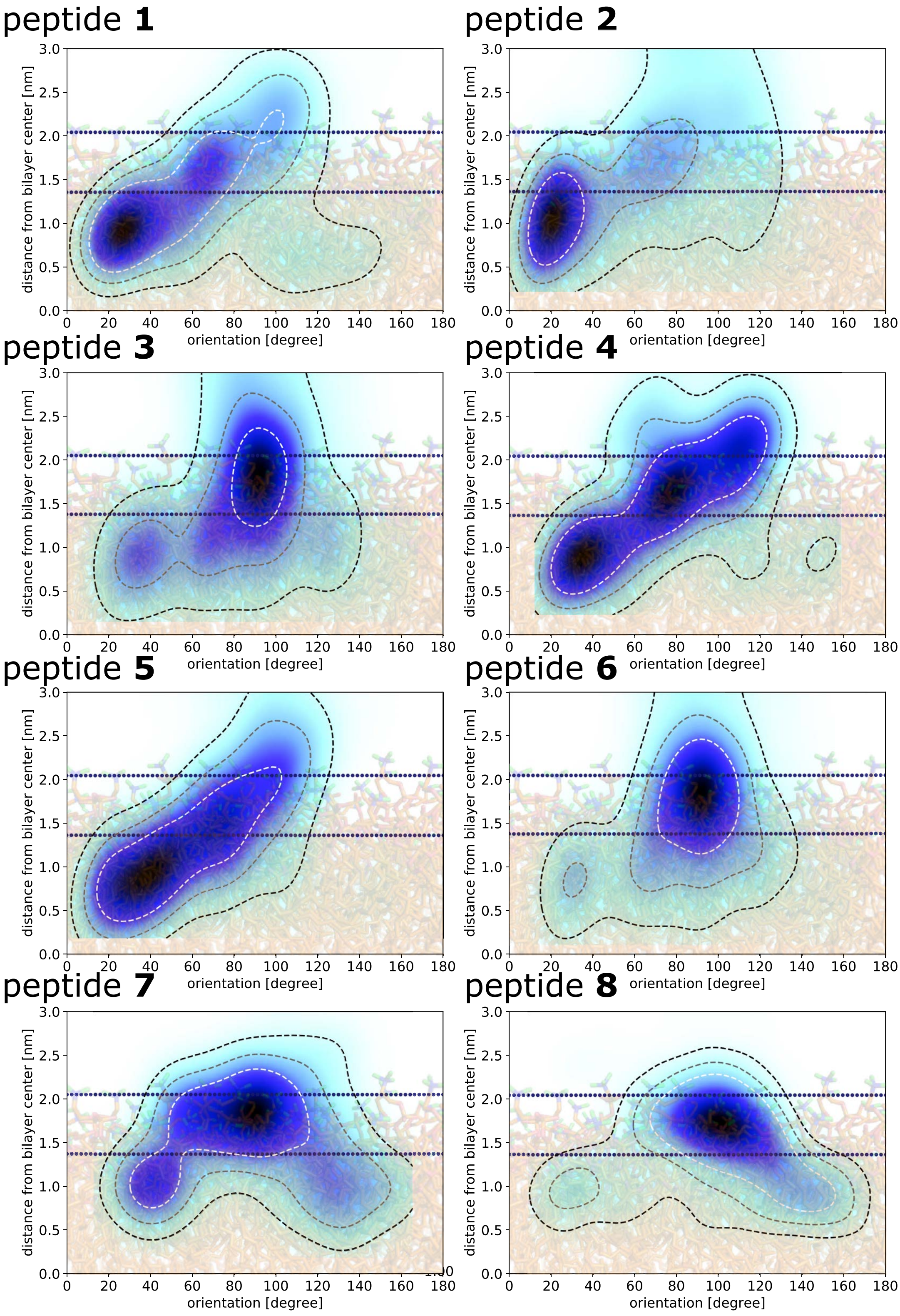

Figure S5: Positional phase space of CDPs **1**-**8** in the open conformation. The coordinates of the peptides are projected onto its distance from the bilayer center and its orientation in respect to the membrane. The heatmap shows the distribution of the simulation time spent in this phase space. Darker color corresponds to more simulation time.

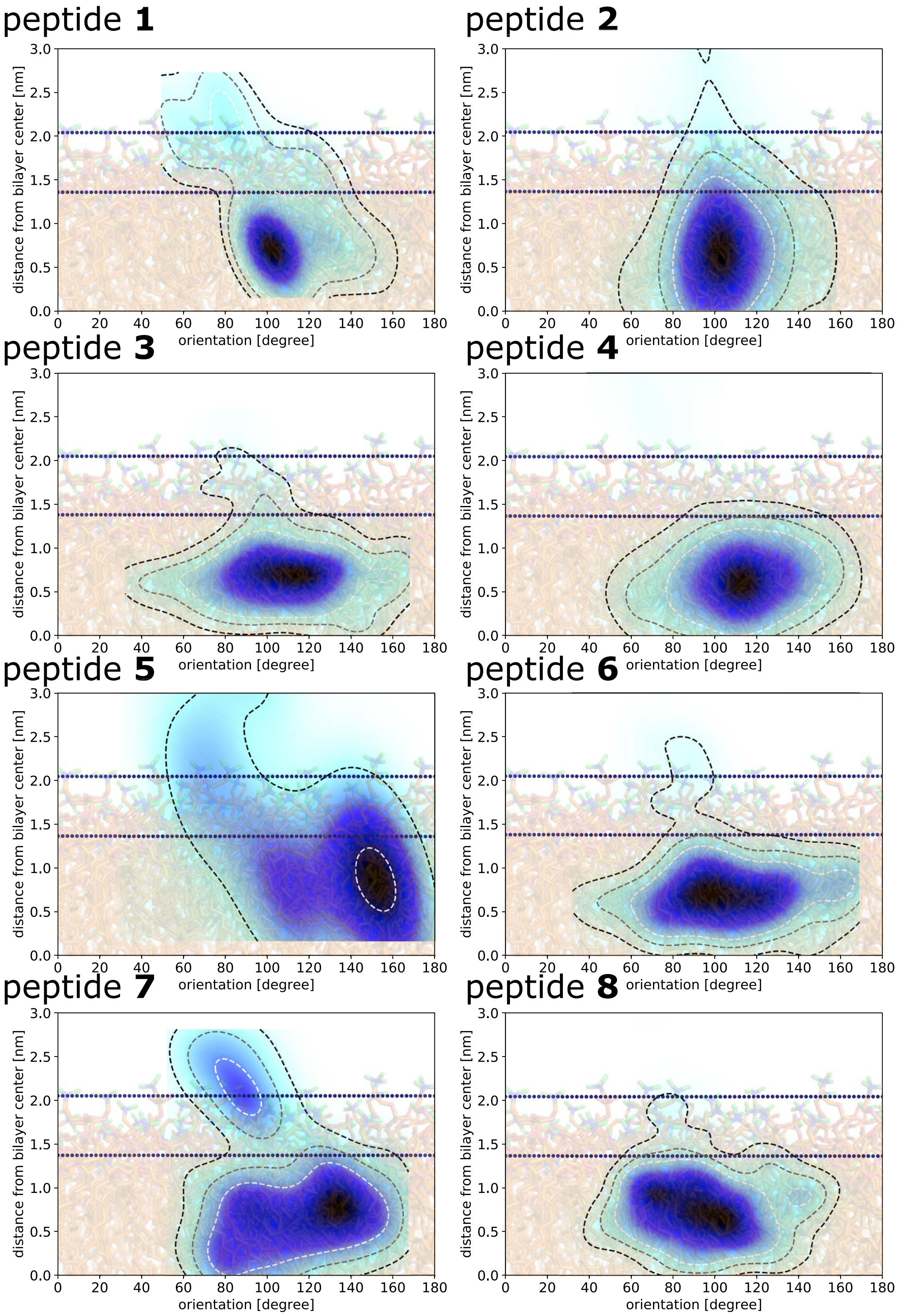

Figure S6: Positional phase space of CDPs **1**-**8** in the closed conformation. The coordinates of the peptides are projected onto its distance from the bilayer center and its orientation in respect to the membrane. The heatmap shows the distribution of the simulation time spent in this phase space. Darker color corresponds to more simulation time.

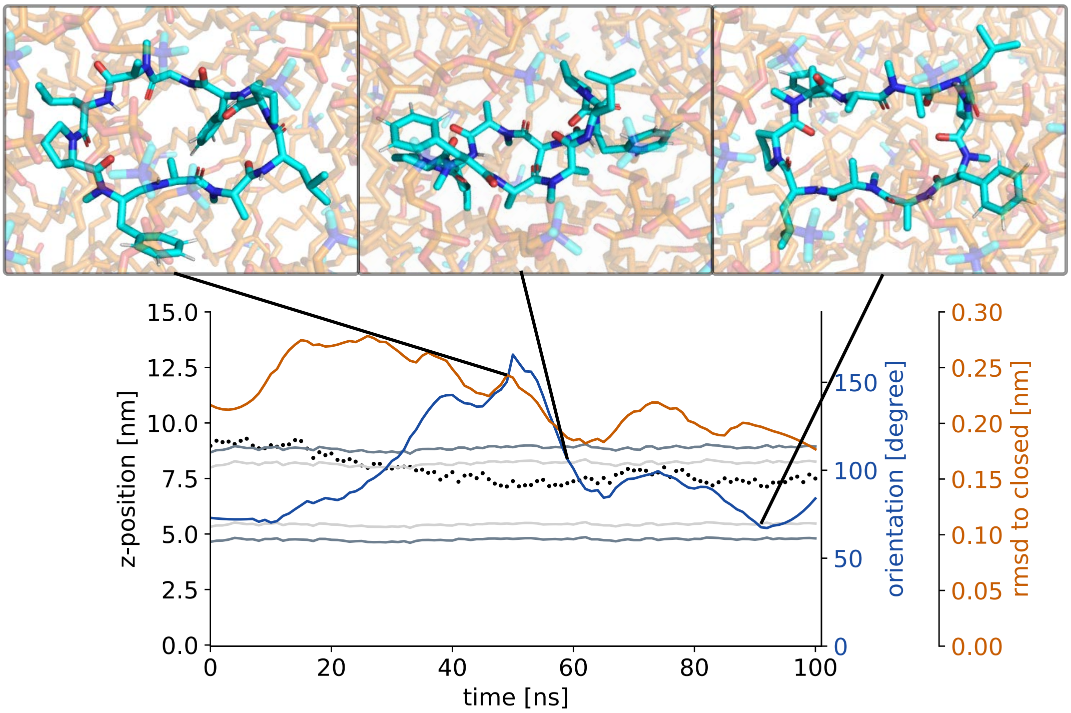

Figure S7: Rotation of CDP **6** from orientation B to A in the open conformation. The peptide rotates along its major axis defined by the peptide backbone. Simulation snapshots of points of interest are shown. The z-position of the CDP is indicated with black dots. The position of the headgroup region and lipid tails are indicated with dark grey and light grey lines, respectively. The angle between the normal vectors of the peptide and the membrane is shown in blue. The RMSD with respect to the ‘closed’ conformation of the CDP is shown in orange.

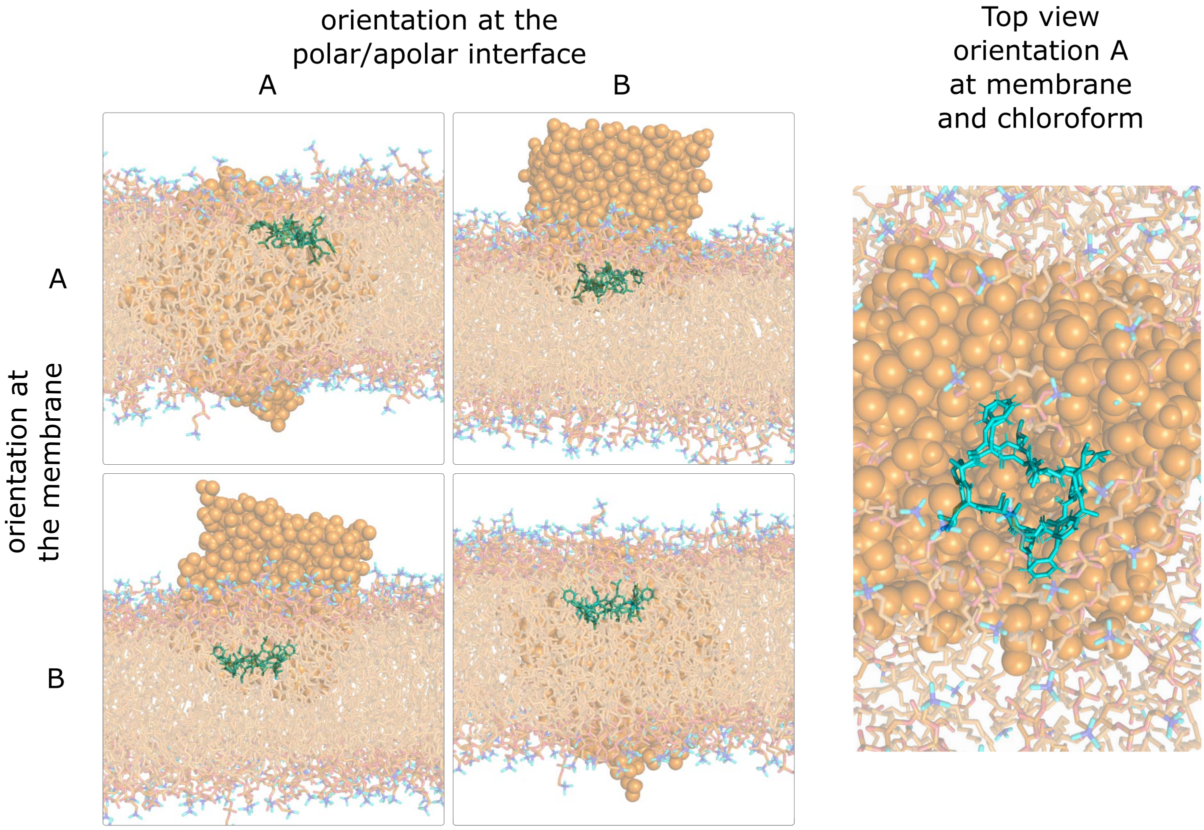

Figure S8: Comparison of the orientations and conformations found at a polar/apolar water/chloroform interface and at the POPC membrane. Orientations and conformations at the polar/apolar interface were taken from Ref. 6. Chloroform molecules are shown as orange balls. Water molecules are omitted for visual clarity. Orientation A at the interface agrees well with orientation A at the membrane. Similarly, orientation B at the interface agrees with orientation B at the membrane. This indicates that the peptides adopt comparable orientations and conformations in both systems.

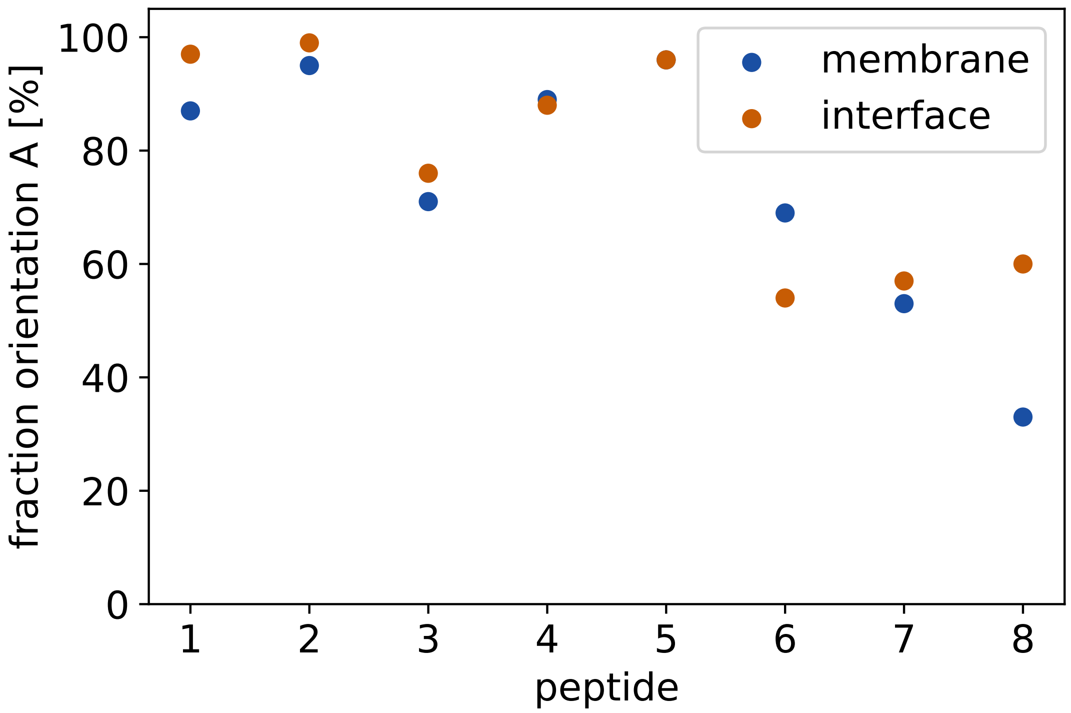

Figure S9: Fraction of simulation frames in orientation A at a water/chloroform interface (orange) and the POPC membrane (blue). The values for the membranes were taken from Table 2 in the main text. The values for the water/chloroform interface were taken from Ref. 6.

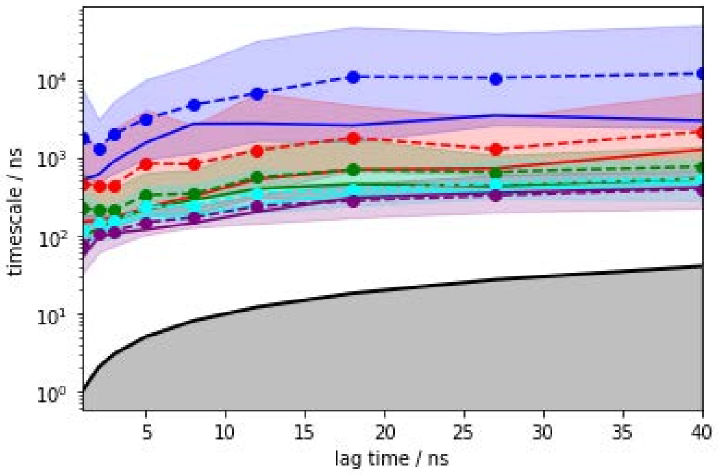

Figure S10: Implied timescales of the MSM of CDPs **4**

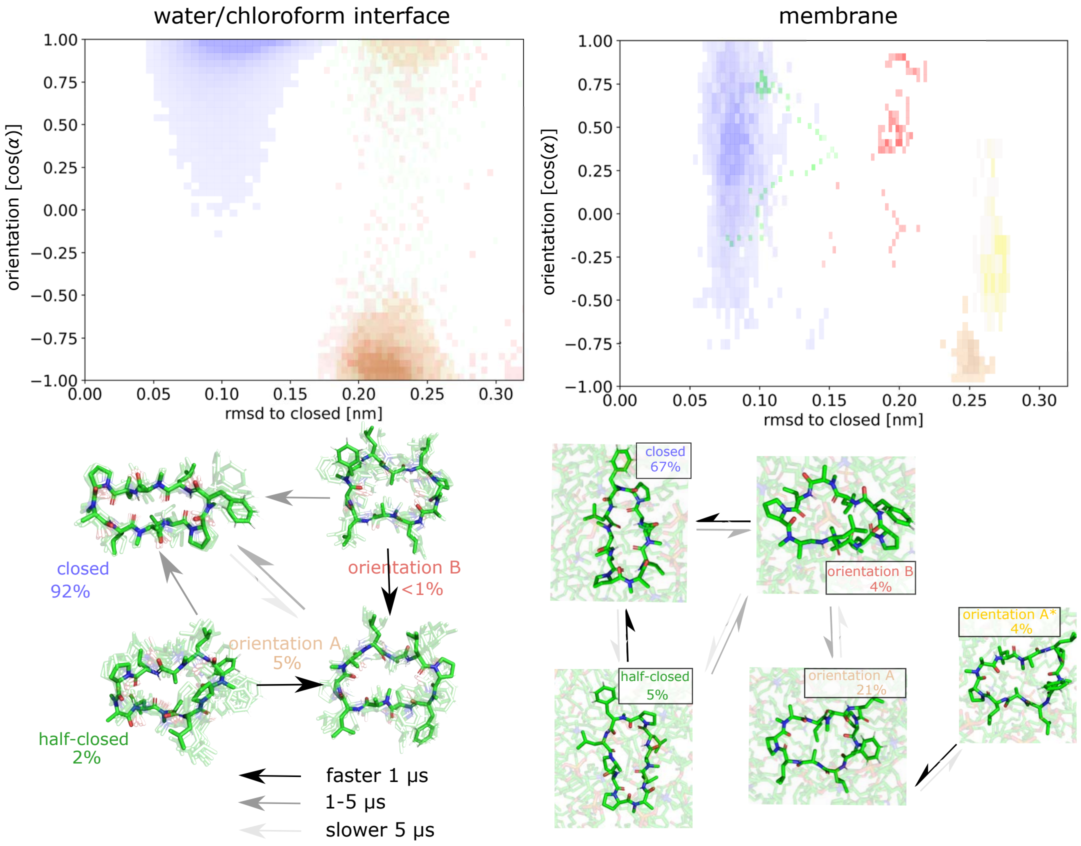

Figure S11: MSM of CDP **4** in the water/chloroform system (left) and at the membrane (right). Top panel: Metastable state assignment mapped to the orientation feature and the RMSD with respect to the closed state. Bottom panel: Representative members of the metastable states, their equilibrium populations and mean first passage times between them.

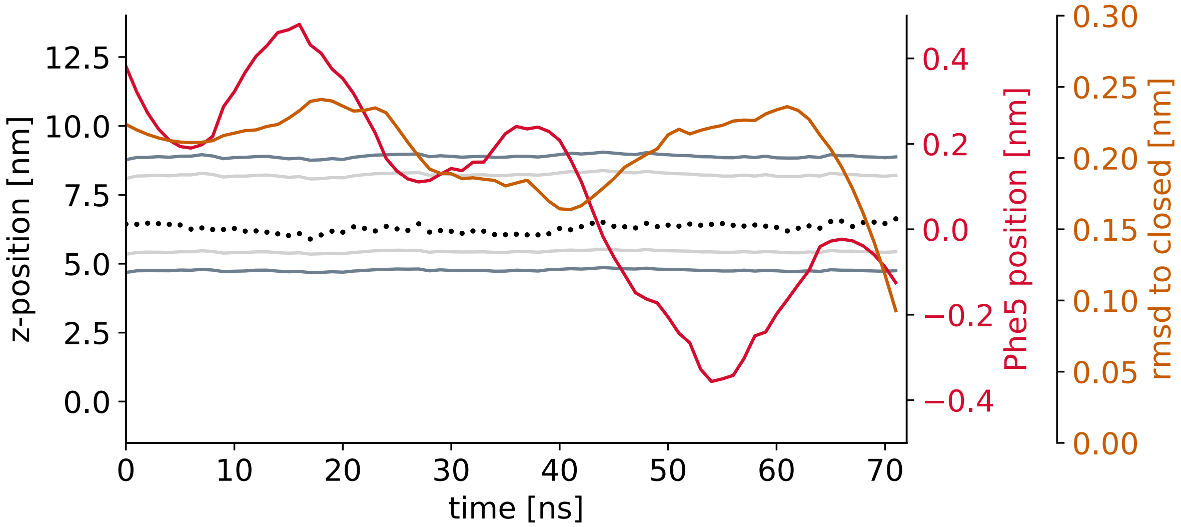

Figure S12: Phenylalanine can act as a 'lock' that prevents interconversion from the open to the closed conformation. A bias was applied on the ψ backbone torsional angle of phenylalanine in the position 5 to pull it from the 'locked' to the unlocked position. After unlocking, the peptide started closing. The closing of the peptide is traced by the RMSD with respect to the closed reference conformation (orange line). The red line indicates the relative position of phenylalanine residue 5 with respect the the ring plane of the peptide. The dotted line indicates the z-position of the peptide. The membrane position is shown as a reference (grey).

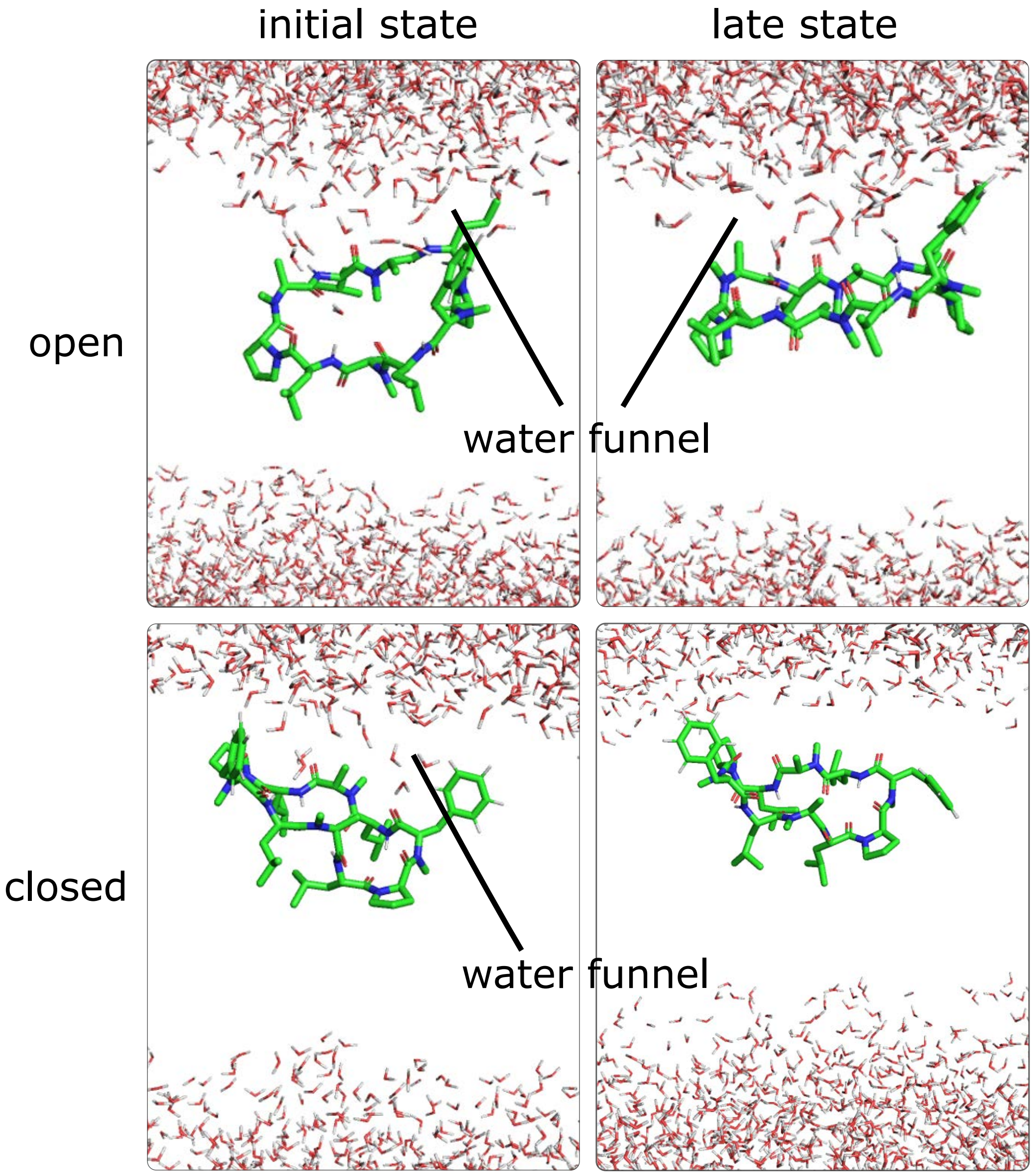

Figure S13: Water interactions of the peptide inside in the membrane in the open and closed orientation. The lipid molecules are not shown for visual clarity. In the 'open' conformation, the peptide stays in contact with the aqueous phase through a water funnel. In the closed conformation, the peptide loses all water contacts and fully emerges into the membrane.

3 Additional Tables

Table S1: Summary of the observed unbiased permeation steps across CDPs **1-8**.

CDP	1	2	3	4	5	6	7	8
Anchoring (open)	2	0	1	2	4	0	2	0
Anchoring (closed)	0	0	1	0	1	0	0	0
Opening	1	0	0	5	0	1	2	2
Closing	0	0	1	2	1	1	1	7
Half-closing	0	1	0	2	2	0	3	5
Leaflet crossing permanent	0	2	1	0	0	0	1	1
Leaflet crossing transient	0	2	1	0	0	0	0	1

References

[1] Gautier, R.; Bacle, A.; Tiberti, M. L.; Fuchs, P. F.; Vanni, S.; Antonny, B. PackMem: A Versatile Tool to Compute and Visualize Interfacial Packing Defects in Lipid Bilayers. *Biophys. J.* **2018**, *115*, 436–444.

[2] Pande, V. S.; Beauchamp, K.; Bowman, G. R. Everything You Wanted to Know About Markov State Models But Were Afraid to Ask. *Methods* **2010**, *52*, 99–105.

[3] Scherer, M. K.; Trendelkamp-Schroer, B.; Paul, F.; Pérez-Hernández, G.; Hoffmann, M.; Plattner, N.; Wehmeyer, C.; Prinz, J.-H.; Noé, F. PyEMMA 2: A Software Package for Estimation, Validation, and Analysis of Markov Models. *J. Chem. Theory Comput.* **2015**, *11*, 5525–5542.

[4] Pérez-Hernández, G.; Paul, F.; Giorgino, T.; De Fabritiis, G.; Noé, F. Identification of Slow Molecular Order Parameters for Markov Model Construction. *J. Chem. Phys.* **2013**, *139*, 015102.

[5] Deuflhard, P.; Weber, M. Robust Perron Cluster Analysis in Conformation Dynamics. *Linear Algebra Appl.* **2005**, *398*, 161–184.

[6] Linker, S. M.; Schellhaas, C.; Ries, B.; Roth, H.-J.; Fouché, M.; Rodde, S.; Riniker, S. Polar/apolar Interfaces Modulate the Conformational Behavior of Cyclic Peptides With Impact on Their Passive Membrane Permeability. *RSC Adv.* **2022**, *12*, 5782–5796.
